# Discovery of 5,7-Dimethoxy-2-(3,4,5-trimethoxyphenoxy)-chromen-4-one with Lipid Lowering Effects in Hepatocytes

**DOI:** 10.3390/ph15040449

**Published:** 2022-04-04

**Authors:** Yi-Han Chang, Chia-Hung Yen, Chih-Chung Lai, Hsuan-Yu Lai, Hsin-Yi Hung

**Affiliations:** 1 School of Pharmacy, National Cheng Kung University Hospital, College of Medicine, National Cheng Kung University, Tainan 701, Taiwan; s68081503@mail.ncku.edu.tw (Y.-H.C.); hsuanyu1113@gmail.com (H.-Y.L.); 2 Graduate Institute of Natural Products, Kaohsiung Medical University, Kaohsiung 807, Taiwan; chyen@kmu.edu.tw (C.-H.Y.); t139725846aa@yahoo.com.tw (C.-C.L.); 3 National Natural Product Libraries and High-Throughput Screening Core Facility, Kaohsiung Medical University, Kaohsiung 807, Taiwan; 4 Drug Development and Value Creation Research Center, Kaohsiung Medical University, Kaohsiung 807, Taiwan; 5 Department of Medical Research, Kaohsiung Medical University Hospital, Kaohsiung 807, Taiwan

**Keywords:** capillarisin, 2-phenoxychromone, NAFLD and NASH, Huh7 cells, lipid accumulation, PGC1α

## Abstract

The population with nonalcoholic fatty liver disease (NAFLD) and nonalcoholic steatohepatitis (NASH) is increasing. However, no medicine is indicated for treating these diseases clinically nowadays. Therefore, there is an urgent need to develop a new drug to overcome NAFLD and NASH. Capillarisin, a 2-phenoxychromone originating from *Artemisia capillaris* Thunb., is well-known for its liver-protective effects. As a result, a series of 2-phenoxychromones was prepared and evaluated for its protective activity against lipid droplet formation in oleic acid (OA)-treated Huh7 cells by means of high-content screening. In the light of the results, the compounds with trimethoxy groups on the phenyl ring possessed better inhibitory properties against lipid accumulation in Huh7 cells, compared to other functional groups on the same ring. Nonetheless, the compounds with a hydroxy group at the C-5 position of the chromone exhibited apparent cytotoxicity. Finally, the active compound, 5,7-dimethoxy-2-(3,4,5-trimethoxyphenoxy)-chromen-4-one (**7e**), with an IC_50_ value of 32.2 ± 2.1 μM against lipid accumulation and no significant cytotoxicity, reduced the accumulation of lipid droplets by up-regulating peroxisome proliferator-activated receptor gamma coactivator 1α (PGC1α) to facilitate the catabolism of fat, which shows promise for further optimization to manage NAFLD and NASH.

## 1. Introduction

Nonalcoholic fatty liver disease (NAFLD) is one of the leading causes of liver disease in the world; it has been estimated that over 25% of people are suffering from this disorder [1]. NAFLD is characterized by an overaccumulation of triglycerides (TG) in the liver, which may progress to nonalcoholic steatohepatitis (NASH), the condition where fat induces lipotoxicity and inflammation, causing harm to hepatocytes. Furthermore, if NASH is not well-controlled, chances are that it will progress to liver cirrhosis, liver failure, and even hepatocellular carcinoma (HCC) [2]. Despite such risks, few drugs are currently used to manage NAFLD and NASH, and no medicine has been indicated for their clinical treatment. Therefore, it is extremely urgent to develop a new drug for treating NAFLD and NASH [3].

**Figure 1:**
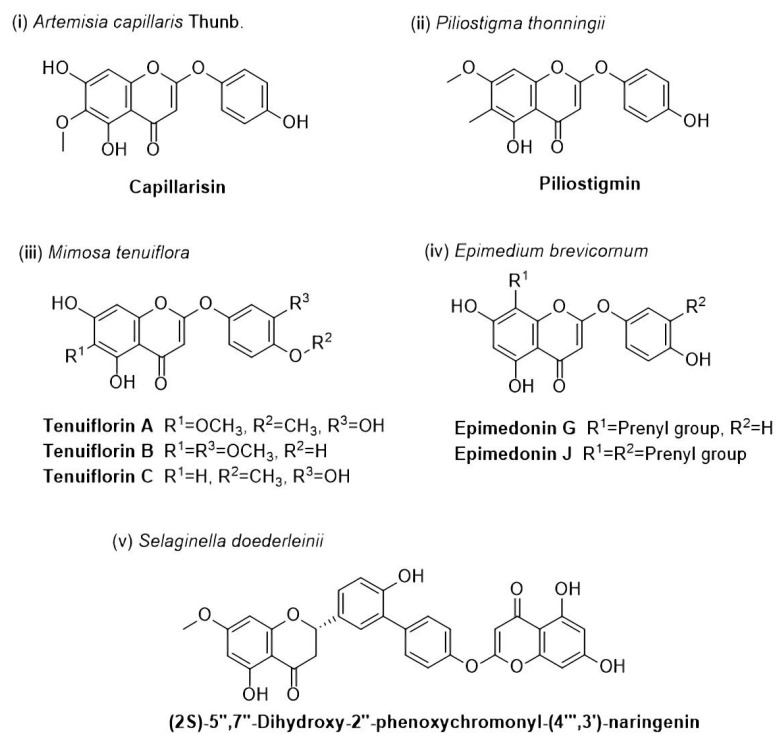
Examples of 2-phenoxychromones in natural products.

The structure of 2-phenoxychromone is unique since it contains an oxygen atom bridging a phenyl group and the C-2 position of a chromone ring. 2-Phenoxychromone compounds are uncommon in natural products—they have been reported to exist only in *Artemisia capillaris* Thunb. [4], *Piliostigma thonningii* [5], *Mimosa tenuiflora* [6], *Epimedium brevicornum* [7], and *Selaginella doederleinii* [8] (Figure 1). Among these plants, *A. capillaris* Thunb. (Yin-Chen-Hao), a Traditional Chinese Medicine (TMC), is well-known for its liver-protective effects [9]. *A. capillaris* Thunb. is the principal medicine in several TMC formulae, such as Yin-Chen-Hao-Tang [10] and Yin-Chen-Wulin-San [11] used in treating liver diseases, such as hepatic injury and jaundice. In addition, a study has demonstrated that *A. capillaris* Thunb. extracts had hypolipidemic and anti-apoptotic effects on HepG2 cells treated with free fatty acids [12]. Isolated from *A. capillaris* Thunb. (Yin-Chen-Hao), capillarisin, one of the 2-phenoxychromones, has been demonstrated to have not only choleretic effects in rats [4] but also anti-oxidative and anti-apoptotic effects in rat primary hepatocytes treated with glycochenodeoxycholic acid or tert-butylhydroperoxide [13,14]. It has also been shown to reduce inflammatory responses in lipopolysaccharide-induced macrophages, which is an important pathophysiological factor that mediates the progression of many diseases [15]. Apart from the effects observed in natural products, some synthetic derivatives have also been reported to exert anti-inflammatory effects by suppressing the generation of superoxide anions by human neutrophils [16]. In addition, sulfur-containing analogs have been observed to be potent inhibitors of aldose reductase (AR) [17]. The research presented above suggests that capillarisin and 2-phenoxychromones possess liver-protective properties.

In order to effectively deal with NAFLD and NASH, it may be beneficial to target their three critical pathological factors: inflammation, insulin resistance, and the overaccumulation of triglycerides in hepatocytes [2]. As mentioned above, capillarisin and its analogs have been reported to possess anti-inflammatory properties and to be capable of overcoming insulin resistance by inhibiting AR. However, there have been no studies indicating that capillarisin or 2-phenoxychromones have hypolipidemic effects on hepatocytes. As multi-functional liver-protective agents, capillarisin and its analogs may also have therapeutical effects on NAFLD and NASH by reducing lipid accumulation in the liver. Therefore, this study aimed to determine whether capillarisin and its derivatives exert liver-protective effects by decreasing fat accumulation in hepatocytes. To this end, a series of 2-phexnoychormones was synthesized and high-content screening was used to evaluate their activity against lipid accumulation in oleic acid (OA)-treated Huh7 cells. In addition, as the overaccumulation of TG in hepatocytes results from the imbalance of free fatty acid uptake and metabolism, the expression of several genes was investigated to determine the possible mechanisms involved in the active compound inhibiting lipid accumulation.

## 2. Results and Discussion

### 2.1. Chemistry

**Scheme 1:**
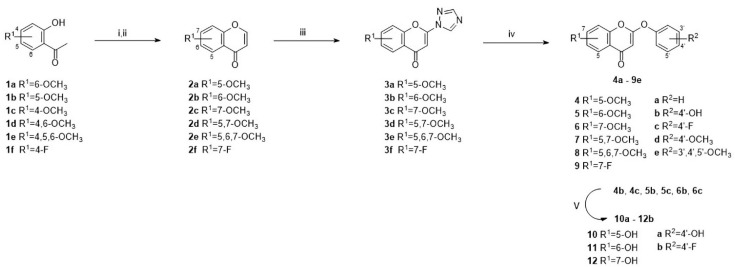
Synthesis of 2-phenoxychromones (**4a**–**12b**). Reagents and conditions: (**i**) DMF-DMA, >100 °C; (**ii**) HCl, DCM, 40 °C; (**iii**) I_2_, 1,2,4-triazole, K_2_CO_3_, DMF, 80 °C; (**iv**) phenolic compounds (**3a**–**3f**), K_2_CO_3_ or Cs_2_CO_3_, DMF, 80 °C; (**v**) BBr_3_ (1 M in DCM), 0 °C → rt.

The general synthetic procedure is demonstrated in Scheme 1. Initially, 2-hydroxyacetophenones which were substituted with methoxy or fluoro groups (**1a**–**1f**), were converted into the corresponding enaminoketones under refluxing conditions, in the presence of *N*, *N*-dimethylformamide dimethyl acetal (DMF-DMA). The HCl-mediated ring closure of the enaminoketones produced chromones (**2a**–**2f**). Catalyzed by 1,2,4-triazole, the resulting chromones reacted with molecular iodine to form 3-iodo-chromone under basic conditions. These reaction intermediates were then attacked by 1,2,4-triazole at the C-2 position and simultaneously underwent dehydroiodination at the C-3 position to produce substituted 2-(1*H*-1,2,4-triazol-1-yl)-chromen-4-ones (**3a**–**3f**). Functioning as a leaving group, the triazole group in **3a**–**3f** was attacked by different phenolic compounds, which is a base-mediated nucleophilic substitution reaction, to produce **4a**–**9e** [18]. To acquire compounds with hydroxyl groups at the chromone ring (**10a**–**12b**), the corresponding compounds were demethylated with boron tribromide.

### 2.2. Structure-Activity Relationship

By means of high-content screening, capillarisin and 2-phenoxychromone derivatives were examined for the accumulation of lipid droplets as well as viability in Huh7 cells (Table 1).

Surprisingly, capillarisin showed no activity against lipid accumulation in hepatocytes and slight cytotoxicity at the tested concentration. In order to determine the structure-activity relationship (SAR) of every functional group on capillarisin, several mono-substituted analogs on the chromone ring (R^1^), with the 4′-hydroxyl group retained on the phenyl ring, were synthesized, and their activity was investigated.

As there is only one methoxy group in the structure of capillarisin, we were curious about the effect of the methoxy substitution. Nevertheless, regardless of the position of the methoxy groups (**4b**, **5b**, and **6b**), neither lipid accumulation nor cell viability decreased. In contrast, lipid droplets tended to slightly increase in Huh7 cells. On the other hand, whereas analogs with a hydroxy group (**10a**, **11a** and **12a**) still did not decrease lipid amounts, compound **10a**, with a hydroxy group at the 5-position, displayed significant cytotoxicity, with a Huh7 cell viability of 63.2 ± 22.3% compared to the control group. This result remained consistent for compound **10b** and capillasirin, both of which have a hydroxy group at the same place. Expanding the range of substitutions to include 5,7-dimethoxy groups (**7b**), 5,6,7-trimethoxy groups (**8b**), or 7-fluoro group (**9b**) did not improve the activity under investigation.

Since the chromone ring did not contribute to any anti-lipid accumulation effects, the focus was next put on the phenoxy ring. As a hydroxy group at the 4′-position of capillarisin is a hydrogen bond donor, analogs with a hydrogen bond acceptor, such as a methoxy group (**4d**, **5d**, **6d**, **7d**, **8d**, and **9d**) or a fluoro group (**5c**, **6c**, **7c**, **8c**, **9c**, **10b**, **11b**, and **12b**) at the same place were prepared, and their activity was examined. None of them exhibited anti-lipid accumulation effects, and **10b** showed prominent cytotoxicity. Compounds without any substitutions on the phenyl ring (**5a**, **6a**, **7a**, **8a**, and **9a**) did not perform better, and some of them (**6a** and **7a**) even caused the phenyl ring to deteriorate.

**Figure 2:**
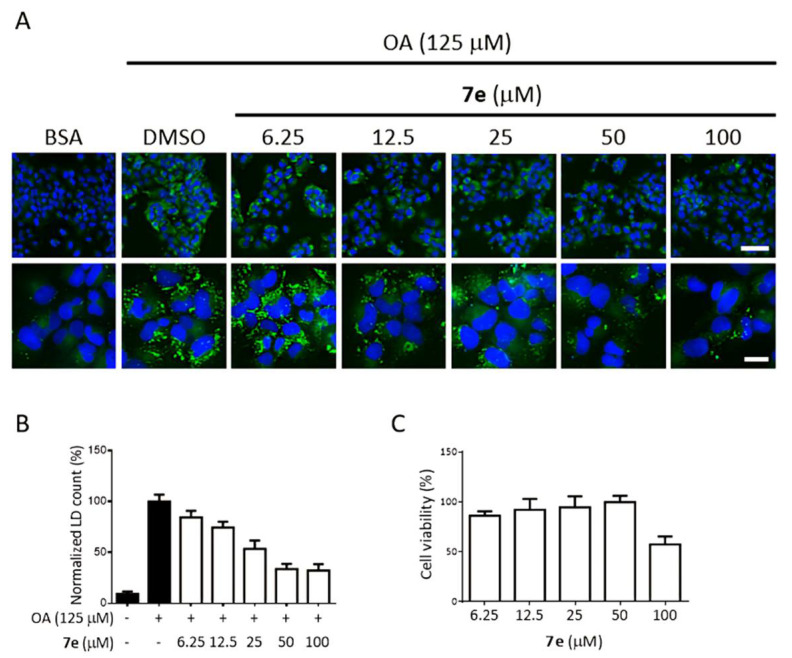
**7e** reduced LD accumulation in a dose-dependent manner. (**A**, **B**) Huh7 cells were treated with either bovine serum albumin (BSA) or BSA-conjugated oleic acid (OA) (125 mM) with the indicated concentrations of **7e** for 16 h. Cells were then fixed with paraformaldehyde and stained with 2 μg/mL of Hoechst 33342 and 1 μg/mL of BODIPY^®^ 493/503. (**A**) Representative images. Scale of upper row: 100 μm. Scale of lower row: 25 μm. (**B**) Quantification results. The IC_50_ of **7e** was 32.2 ± 2.1 μM. (**C**) The cytotoxicity of **7e** in Huh7 cells. Huh7 cells were treated with a series of concentrations of **7e** for 72 h. Cell viability was measured by alamarBlue^®^ reagent according to the manufacturer’s protocol. The results showed that the CC_50_ of **7e** was higher than 100 μM.

With hydrogen bonds having been shown not to play a role in the activity under investigation, electron density was speculated to be the dominant factor. As expected, compounds with 3′,4′,5′-trimethoxy groups (**5e**, **7e**, and **8e**) had a great impact on the amount of lipid droplets in Huh7 cells. This was not the case for **6e** and **9e**, which implied that mono-substitution at the 7-position of the chromone ring did not promote the activity. In addition, **5e** showed some cytotoxicity in Huh7 cells at the tested concentration. As a result, **7e**, the active compound with the IC_50_ value of 32.2 ± 2.1 μM (Figure 2A, B) against lipid accumulation and without cytotoxicity (CC_50_ > 100 μM) (Figure 2C), was further investigated for *in vitro* pharmacological mechanisms.

### 2.3. In Vitro Pharmacological Mechanisms

In order to determine whether compound **7e** reduced lipid levels in Huh7 cells by suppressing the cell uptake of fatty acids to form lipid droplets, or by facilitating the metabolism of fat, Huh7 cells were co-treated with 125 μM of OA and **7e** at the concentrations of 25, 50 and 100 μM for 16 h. RT-qPCR was applied to the RNA of harvested Huh7 cells in order to analyze the expression of several genes.

**Figure 3:**
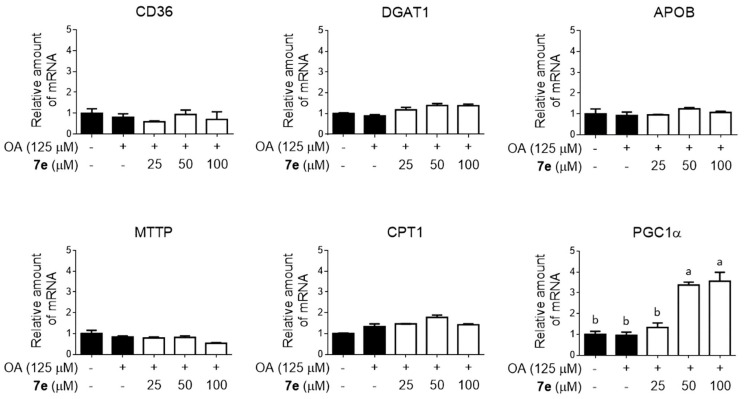
The effects of **7e** on lipid metabolism gene expression. Huh7 cells were treated with either bovine serum albumin (BSA) or BSA-conjugated oleic acid (OA) (125 mM) with the indicated concentrations of **7e** for 16 h. RT-qPCR was used to determine the expression profile of lipid metabolism-associated genes: Lipid entry, CD36; TG formation, DGAT1; β-oxidation, CPT1; lipid secretion, APOB and MTTP; and mitochondria biogenesis, PGC1α. Statistical analysis was performed using ANOVA, and different superscript letters (a and b) indicate statistically significant differences (*p* < 0.05).

The results are presented in Figure 3. As the major source of TG stored in hepatocytes originates in peripheral adipose tissues in people with NAFLD [19], a study [20] has investigated the expression of gene CD36, which encodes the translocase that facilitates the ingestion of fatty acids into hepatocytes [21], and the expression of gene diglyceride acyltransferase 1 (DGAT1), which encodes the enzyme that determines the rate of triglyceride assembly from absorbed free fatty acid. However, the absence of a significant difference in the expression of CD36 and DGAT1 in **7e**-treated groups compared with OA-treated groups indicates that **7e** did not reduce the amount of lipid droplets by inhibiting OA uptake. In addition, **7e** did not remove lipids by secreting very low density lipoproteins (VLDL) from hepatocytes, since the expression of both apolipoprotein B (APOB) and microsomal triglyceride transfer protein (MTTP) was comparable between **7e**-treated groups and control groups. However, the expression of a gene that encodes peroxisome proliferator-activated receptor gamma coactivator 1α (PGC1α), which participates in the biogenesis of mitochondria by regulating the activity of many genes, such as nuclear respiratory factor 1 (NRF1), and thereby increases the oxidation of hepatic triglycerides [22], was significantly induced by **7e** in a dose-dependent manner. There was also a slight increase in the expression of the carnitine palmitoyltransferase I (CPT1) gene, which encodes the rate-limiting enzyme for fatty acid β-oxidation [23]. These results revealed that **7e** reduced the number of lipid droplets in Huh7 cells by improving the catabolism of fat. In sum, compound **7e** shows promise as a potential agent in preventing NAFLD or NASH.

## 3. Materials and Methods

### 3.1. Chemistry

All chemicals (reagent grade) were purchased from Sigma-Aldrich (Burlington, MA, USA), Alfa Aesar (Ward Hill, MA, USA), and Merck (Burlington, MA, USA) without being further purified. Reaction progress was monitored by thin layer chromatography (TLC) with precoated silica gel 60 F254 plates of a thickness of 0.25 mm (Merck), and spots were detected with UV light (254 nm and/or 360 nm). Column chromatography was performed on silica gel (70–230 mesh and 230–400 mesh). ^1^H- and ^13^C-NMR spectra were recorded on a Bruker AMX-400 spectrometer, using a deuterated solvent as the internal standard. Standard pulse sequences and parameters were used for the NMR experiments, and all chemical shifts are reported in parts per million (ppm, δ). Splitting patterns were designed as s, singlet; d, doublet; dd, doublet of doublet; ddd, doublet of doublet of doublet; t, triplet; m, multiplet; and br, broadband. The purity of all compounds was confirmed to be higher than 95% by means of analytical HPLC performed with a Shimadzu LC-20AT system and an SPD-20A UV detector. High-resolution mass spectra were measured in the instrument center of National Sun Yat-sen University (Bruker FT-MS SolariX). Capillarisin was provided by courtesy of Prof. Tian-Shung Wu’s lab and was isolated from *A. capillaris* [24].

#### 3.1.1. General Procedure for the Synthesis of Substituted 4H-chromen-4-one (**2a**–**2f**)

One equivalent of substituted 2-hydroxy-methoxyacetophenone (**1a**–**1f**) was dissolved in 1.5 equivalents of DMF-DMA and stirred at over 100 °C for between 10 min to 2 h. The mixture was allowed to cool down at room temperature until crystals formed, which were washed with hexane. The precipitate was dissolved in dichloromethane (DCM) with concentrated HCl, and the mixture was stirred at 40 °C. The progress of the reaction was checked by TLC. After completion, the reaction mixture was poured into a separatory funnel and extracted with DCM. The organic layers were combined, dried over anhydrous MgSO_4_, filtered, and concentrated in vacuo. The residue was purified by column chromatography.

##### 5-Methoxychromen-4-one (**2a**) 

An amount of 400.0 mg (2.4 mmol) of 2-hydroxy-6-methoxyacetophenone (**1a**) was dissolved in DMF-DMA (0.5 mL, 3.6 mmol) and stirred at over 100 °C for 120 min. The mixture was allowed to cool down at room temperature until crystals formed, which were washed with hexane. The precipitate was dissolved in DCM (11.0 mL) with concentrated HCl (1.4 mL), and the mixture was stirred at 40 °C. The progress of the reaction was checked by TLC. After 30 min, the reaction mixture was poured into a separatory funnel and extracted with DCM (15 mL × 3). The organic layers were combined, dried over anhydrous MgSO_4_, filtered, and concentrated in vacuo. The residue was purified by column chromatography (silica gel, hexane: ethyl acetate = 1:1, *v*/*v*) to produce **2a** as a brown syrup (405.9 mg, yield: 96.0%) [25].

##### 6-Methoxychromen-4-one (**2b**)

An amount of 498.8 mg (3.0 mmol) of 2-hydroxy-5-methoxyacetophenone (**1b**) was dissolved in DMF-DMA (0.6 mL, 4.5 mmol) and stirred at over 100 °C for 30 min. The mixture was allowed to cool down at room temperature until crystals formed, which were washed with hexane. The precipitate was dissolved in DCM (11.0 mL) with concentrated HCl (1.6 mL), and the mixture was stirred at 40 °C. The progress of the reaction was checked by TLC. After 20 min, the reaction mixture was poured into a separatory funnel and extracted with DCM (15 mL × 3). The organic layers were combined, dried over anhydrous MgSO_4_, filtered, and concentrated in vacuo. The residue was purified by column chromatography (silica gel, hexane: ethyl acetate = 2:1, *v*/*v*) to produce **2b** as a yellowish solid (505.3 mg, yield: 95.6 %). ^1^H NMR (400 MHz, CDCl_3_) δ 7.85 (d, *J* = 6.0 Hz, 1H), 7.57 (d, *J* = 3.2 Hz, 1H), 7.40 (d, *J* = 9.2 Hz, 1H), 7.26 (dd, *J* = 9.2, 3.2 Hz, 1H), 6.33 (d, *J* = 6.0 Hz, 1H), and 3.89 (s, 3H). ^13^C NMR (100 MHz, CDCl_3_) δ 177.7, 157.1, 155.2, 151.6, 125.6, 124.1, 119.8, 112.3, 105.0, and 56.1 [26].

##### 7-Methoxychromen-4-one (**2c**)

An amount of 498.8 mg (3.0 mmol) of 2-hydroxy-4-methoxyacetophenone (**1c**) was dissolved in DMF-DMA (0.6 mL, 4.5 mmol) and stirred at over 100 °C for 10 min. The mixture was allowed to cool down at room temperature until crystals formed, which were washed with hexane. The precipitate was dissolved in DCM (11.0 mL) with concentrated HCl (1.6 mL), and the mixture was stirred at 40 °C. The progress of the reaction was checked by TLC. After 30 min, the reaction mixture was poured into a separatory funnel and extracted with DCM (15 mL × 3). The organic layers were combined, dried over anhydrous MgSO_4_, filtered, and concentrated in vacuo. The residue was purified by column chromatography (silica gel, hexane: ethyl acetate = 1:1, *v*/*v*) to produce **2c** as an orange solid (465.1 mg, yield: 88.0 %). ^1^H NMR (400 MHz, CDCl_3_) δ 8.11 (d, *J* = 9.2 Hz, 1H), 7.78 (d, *J* = 6.0 Hz, 1H), 6.97 (dd, *J* = 9.2, 2.4 Hz, 1H), 6.84 (d, *J* = 2.4 Hz, 1H), 6.28 (d, *J* = 6.0 Hz, 1H), and 3.90 (s, 3H) [27].

##### 5,7-Dimethoxychromen-4-one (**2d**)

2-Hydroxy-4,6-dimethoxyacetophenone (**1d**) was obtained in accordance with previously applied processes [28] and produced a white solid (yield: 95.6 %); 320.0 mg (1.6 mmol) of **1d** were dissolved in DMF-DMA (0.3 mL, 2.4 mmol) and stirred at over 100 °C for 10 min. The mixture was allowed to cool down at room temperature until crystals formed, which were washed with hexane. The precipitate was dissolved in DCM (6.5 mL) with concentrated HCl (0.8 mL), and the mixture was stirred at 40 °C. The progress of the reaction was checked by TLC. After 30 min, the reaction mixture was poured into a separatory funnel and extracted with DCM (10 mL × 3). The organic layers were combined, dried over anhydrous MgSO_4_, filtered, and concentrated in vacuo. The residue was purified by column chromatography (silica gel, hexane: ethyl acetate = 1:2, *v*/*v*) to produce **2d** as an orange solid (300.9 mg, yield: 91.2%) [29].

##### 5,6,7-Trimethoxychromen-4-one (**2e**)

6-Hydroxy-2,3,4-trimethoxyacetophenone (**1e**) was obtained in accordance with previously applied processes [30] and produced a yellowish solid (yield: 55.7 %); 1000.0 mg (4.4 mmol) of **1e** were dissolved in DMF-DMA (0.9 mL, 6.6 mmol) and stirred at over 100 °C for 30 min. The mixture was allowed to cool down at room temperature until crystals formed, which were washed with hexane. The precipitate was dissolved in DCM (18.0 mL) with concentrated HCl (2.3 mL), and the mixture was stirred at 40 °C. The progress of the reaction was checked by TLC. After 30 min, the reaction mixture was poured into a separatory funnel and extracted with DCM (20 mL × 3). The organic layers were combined, dried over anhydrous MgSO_4_, filtered, and concentrated in vacuo. The residue was purified by column chromatography (silica gel, hexane: ethyl acetate = 2:3, *v*/*v*) to produce **2e** as a yellowish solid (716.3 mg, yield: 68.9 %). ^1^H NMR (400 MHz, CDCl_3_) δ 7.65 (d, *J* = 6.0 Hz, 1H), 6.67 (s, 1H), 6.18 (d, *J* = 6.0 Hz, 1H), 3.95 (s, 3H), 3.93 (s, 3H), and 3.89 (s, 3H) [31].

##### 7-Fluorochromen-4-one (**2f**)

An amount of 300.0 mg (2.0 mmol) of 2-hydroxy-4-flouroacetophenone (**1f**) was dissolved in DMF-DMA (0.4 mL, 2.9 mmol) and stirred at over 100 °C for 10 min. The mixture was allowed to cool down at room temperature until crystals formed, which were washed with hexane. The precipitate was dissolved in DCM (7.0 mL) with concentrated HCl (1.0 mL), and the mixture was stirred at 40 °C. The progress of the reaction was checked by TLC. After completion, the reaction mixture was poured into a separatory funnel and extracted with DCM (10 mL × 3). The organic layers were combined, dried over anhydrous MgSO_4_, filtered, and concentrated in vacuo. The residue was purified by column chromatography (silica gel, hexane: ethyl acetate = 2:1, *v*/*v*) to produce **2f** as a yellowish solid (318.1 mg, yield: 97.0%). ^1^H NMR (400 MHz, CDCl_3_) δ 8.23–8.19 (m, 1H), 7.83 (d, *J* = 6.0 Hz, 1H), 7.15–7.10 (m, *J* = 2.0 Hz, 2H), and 6.32 (d, *J* = 6.0 Hz, 1H) [32].

#### 3.1.2. General Procedure for the Synthesis of Substituted 2-(1,2,4-triazol-1-yl)chromen-4-one (**3a**–**3f**)

One equivalent of substituted 4*H*-chromen-4-one (**2a**–**2f**) and 2-6 equivalents of 1,2,4-triazole were dissolved in dry DMF to make a 0.2 M solution, followed by the addition of 1.5 equivalents of molecular iodine and anhydrous K_2_CO_3_. The mixture was stirred at 80 °C, and the progress of the reaction was checked by TLC. Upon completion, the mixture was quenched with a sodium thiosulfate solution, extracted with DCM, washed with brine, and dried over anhydrous MgSO_4_. The organic layers were filtered and concentrated in vacuo. The crude product was washed with iced acetone several times to collect the solid. The filtrate was further purified by column chromatography, and the pure compound was combined with the solid as **3a**–**3f.**

##### 5-Methoxy-2-(1,2,4-triazol-1-yl)chromen-4-one (**3a**)

An amount of 469.0 mg (2.7 mmol) of **2a** and 1,2,4-triazole (1105.0 mg, 16.0 mmol) was dissolved in dry DMF (13.3 mL), followed by the addition of molecular iodine (1015.2 mg, 4.0 mmol) and anhydrous K_2_CO_3_ (1838.2 mg, 13.3 mmol). The mixture was stirred at 80 °C, and the progress of the reaction was checked by TLC. After 5.5 h, the mixture was quenched with a sodium thiosulfate solution (15 mL), extracted with DCM (20 mL × 3), and washed with brine (20 mL × 2). The organic layers were combined, dried over anhydrous MgSO_4_, filtered, and concentrated in vacuo. The crude product was washed with iced acetone several times to collect the solid. The filtrate was further purified by column chromatography (silica gel, hexane: ethyl acetate = 2:5, *v*/*v*) and the pure compound was combined with the solid to produce **3a** as a white solid (167.2 mg, yield: 25.8%). ^1^H NMR (400 MHz, CDCl_3_) δ 8.85 (s, 1H), 8.16 (s, 1H), 7.63 (t, *J* = 8.4, 8.4 Hz, 1H), 7.11 (dd, *J* = 8.4, 0.8 Hz, 1H), 6.92 (d, *J* = 8.4 Hz, 1H), 6.78 (s, 1H), and 4.01 (s, 3H). ^13^C NMR (100 MHz, CDCl_3_) δ 177.6, 160.3, 156.3, 154.0, 151.4, 142.0, 134.6, 114.3, 109.6, 108.1, 99.7, and 56.8. HRMS [ESI]^+^ calculated for [C_12_H_9_N_3_O_3_ + H]^+^ 244.0717; found [M + H]^+^ 244.0715.

##### 6-Methoxy-2-(1,2,4-triazol-1-yl)chromen-4-one (**3b**)

An amount of 350.0 mg (2.0 mmol) of **2b** and 1,2,4-triazole (412.3 mg, 6.0 mmol) was dissolved in dry DMF (10 mL), followed by the addition of molecular iodine (758.9 mg, 3 mmol) and anhydrous K_2_CO_3_ (1375.2 mg, 10.0 mmol). The mixture was stirred at 80 °C, and the progress of the reaction was checked by TLC. After 6.5 h, the mixture was quenched with a sodium thiosulfate solution (10 mL), extracted with DCM (20 mL × 3), and washed with brine (20 mL × 2). The organic layers were combined, dried over anhydrous MgSO_4_, filtered, and concentrated in vacuo. The crude product was washed with iced acetone several times to collect the solid. The filtrate was further purified by column chromatography (silica gel, hexane: ethyl acetate = 2:3, *v*/*v*) and the pure compound was combined with the solid to produce **3b** as a white solid (210.5 mg, yield: 43.7%). ^1^H NMR (400 MHz, CDCl_3_) δ 8.88 (s, 1H), 8.17 (s, 1H), 7.61 (d, *J* = 3.2 Hz, 1H), 7.49 (d, *J* = 9.2 Hz, 1H), 7.32 (dd, *J* = 9.2, 3.2 Hz, 1H), 6.88 (s, 1H), and 3.92 (s, 3H). ^13^C NMR (100 MHz, CDCl_3_) δ 177.8, 157.9, 154.0, 153.0, 148.8, 142.1, 124.6, 124.2, 119.0, 105.9, 97.8, and 56.2 [33].

##### 7-Methoxy-2-(1,2,4-triazol-1-yl)chromen-4-one (**3c**)

An amount of 350.0 mg (2.0 mmol) of **2c** and 1,2,4-triazole (412.3 mg, 6.0 mmol) was dissolved in dry DMF (10 mL), followed by the addition of molecular iodine (758.9 mg, 3.0 mmol) and anhydrous K_2_CO_3_ (1375.2 mg, 10.0 mmol). The mixture was stirred at 80 °C, and the progress of the reaction was checked by TLC. After 5 h, the mixture was quenched with a sodium thiosulfate solution (10 mL), extracted with DCM (20 mL × 3), and washed with brine (20 mL × 2). The organic layers were combined, dried over anhydrous MgSO_4_, filtered, and concentrated in vacuo. The crude product was washed with iced acetone several times to collect the solid. The filtrate was further purified by column chromatography (silica gel, hexane: ethyl acetate = 2:3, *v*/*v*) and the pure compound was combined with the solid to produce **3c** as a white solid (310.6 mg, yield: 64.2%). ^1^H NMR (400 MHz, CDCl_3_) δ 8.87 (s, 1H), 8.18 (s, 1H), 8.15 (d, *J* = 8.8 Hz, 1H), 7.01 (dd, *J* = 8.8, 2.0 Hz, 1H), 6.96 (d, *J* = 2.0 Hz, 1H), 6.82 (s, 1H), and 3.95 (s, 3H). ^13^C NMR (100 MHz, CDCl_3_) δ 177.2, 164.8, 155.9, 154.0, 152.8, 142.0, 127.7, 117.5, 115.1, 100.6, 98.3, and 56.2 [33].

##### 5,7-Dimethoxy-2-(1,2,4-triazol-1-yl)chromen-4-one (**3d**)

An amount of 250.0 mg (1.2 mmol) of **2d** and 1,2,4-triazole (250.7 mg, 3.6 mmol) was dissolved in dry DMF (6.0 mL), followed by the addition of molecular iodine (461.9 mg, 1.8 mmol) and anhydrous K_2_CO_3_ (836.2 mg, 6.1 mmol). The mixture was stirred at 80 °C, and the progress of the reaction was checked by TLC. After 5 h, the mixture was quenched with a sodium thiosulfate solution (10 mL), extracted with DCM (20 mL × 3), and washed with brine (20 mL × 2). The organic layers were combined, dried over anhydrous MgSO_4_, filtered, and concentrated in vacuo. The crude product was washed with iced acetone several times to obtain **3d** as a white solid (109.5 mg, yield: 40.2%), which was directly used for the next step.

##### 5,6,7-Trimethoxy-2-(1,2,4-triazol-1-yl)chromen-4-one (**3e**)

An amount of 500.0 mg (2.2 mmol) of **2e** and 1,2,4-triazole (449.0 mg, 6.5 mmol) was dissolved in dry DMF (10.9 mL), followed by the addition of molecular iodine (827.4 mg, 3.3 mmol) and anhydrous K_2_CO_3_ (1451.2 mg, 10.9 mmol). The mixture was stirred at 80 °C, and the progress of the reaction was checked by TLC. After 4 h, the mixture was quenched with a sodium thiosulfate solution (15 mL), extracted with DCM (30 mL × 3), and washed with brine (20 mL × 2). The organic layers were combined, dried over anhydrous MgSO_4_, filtered, and concentrated in vacuo. The crude product was washed with iced acetone several times to collect the solid. The filtrate was further purified by column chromatography (silica gel, hexane: ethyl acetate = 2:3, *v*/*v*) and the pure compound was combined with the solid to produce **3e** as a white solid (287.5 mg, yield: 43.7%). ^1^H NMR (400 MHz, CDCl_3_) δ 8.82 (s, 1H), 8.16 (s, 1H), 6.79 (s, 1H), 6.70 (s, 1H), 3.99 (s, 3H), 3.98 (s, 3H), and 3.92 (s, 3H). ^13^C NMR (100 MHz, CDCl_3_) δ 176.5, 158.3, 154.0, 153.2, 152.4, 151.4, 141.9, 141.4, 112.7, 99.1, 96.2, 62.4, 61.7, and 56.6. HRMS [ESI]^+^ calculated for [C_14_H_13_N_3_O_5_ + H]^+^ 304.0928; found [M + H]^+^ 304.0929.

##### 7-Fluoro-2-(1,2,4-triazol-1-yl)chromen-4-one (**3f**)

An amount of 300.0 mg (1.8 mmol) of **2f** and 1,2,4-triazole (255.6 mg, 3.7 mmol) was dissolved in dry DMF (9.0 mL), followed by the addition of molecular iodine (685.3 mg, 2.7 mmol) and anhydrous K_2_CO_3_ (1257.7 mg, 9.1 mmol). The mixture was stirred at 80 °C, and the progress of the reaction was checked by TLC. After 2 h, the mixture was quenched with a sodium thiosulfate solution (10 mL), extracted with DCM (20 mL × 3), and washed with brine (20 mL × 2). The organic layers were combined, dried over anhydrous MgSO_4_, filtered, and concentrated in vacuo. The crude product was washed with iced acetone several times to collect the solid. The filtrate was further purified by column chromatography (silica gel, hexane: ethyl acetate = 3:2, *v*/*v*) and the pure compound was combined with the solid to produce **3f** as a white solid (148.5 mg, yield: 35.7%). ^1^H NMR (400 MHz, CDCl_3_) δ 8.87 (s, 1H), 8.27 (dd, *J* = 8.8, 6.0 Hz, 1H), 8.18 (s, 1H), 7.29-7.20 (m, 2H), and 6.88 (s, 1H). HRMS [ESI]^+^ calculated for [C_11_H_6_FN_3_O_2_ + H]^+^ 232.0517; found [M + H]^+^ 232.0519.

#### 3.1.3. General Procedure for the Synthesis of Substituted 2-(substituted phenoxy)chromen-4-one (**4a**–**12b**)

One equivalent of substituted 2-(1*H*-1,2,4-triazol-1-yl)chromen-4-one (**3a–3f**) was added into dry DMF or 1,4-dioxane to make a 0.1 M solution. Three equivalents of a phenolic compound were then dissolved in the solution and stirred at 80 °C until clear; 3-6 equivalents of anhydrous K_2_CO_3_ or Cs_2_CO_3_ were added into the mixture at a constant temperature of 80 °C. The progress of the reaction was checked by TLC. After completion, the reaction mixture was diluted with water and partitioned with ethyl acetate. The organic phases were combined and concentrated in vacuo. The crude product was purified by column chromatography to obtain **4a**–**9e**.

For compounds with hydroxyl groups at the chromone ring (**10a-12b**), the corresponding compounds (**4b**, **4c**, **5b**, **5c**, **6b**, **6c**) were dissolved in anhydrous DCM and then treated with three equivalents of boron tribromide (1 M in DCM) dropwise at 0 °C in an inert atmosphere. After 1 h had passed, the resulting solutions were continuously stirred at ambient temperature. The progress of the reaction was checked by TLC. After completion, the reaction mixture was diluted with water and partitioned with ethyl acetate. The organic phases were combined and concentrated in vacuo. The crude product was purified by column chromatography.

##### 5-Methoxy-2-phenoxy-chromen-4-one (**4a**)

An amount of 75.0 mg (0.3 mmol) of **3a** was stirred with phenol (86.6 mg, 0.9 mmol) and Cs_2_CO_3_ (599.5 mg, 1.8 mmol) in 1,4-dioxane (3.0 mL) at 80 °C. After 30 min, the reaction mixture was diluted with water (10.0 mL) and partitioned with ethyl acetate (10 mL × 3). The organic phases were combined and concentrated in vacuo. The crude product was purified by column chromatography (silica gel, hexane: ethyl acetate = 1:3, *v*/*v*) to obtain **4a** as a colorless gum (77.7 mg, yield: 93.4%). ^1^H NMR (400 MHz, CDCl_3_) δ 7.49 (t, *J* = 8.4, 8.4 Hz, 1H), 7.43–7.38 (m, 2H), 7.30–7.25 (m, 1H), 7.15 (dd, *J* = 5.6, 0.8 Hz, 2H), 6.96 (dd, *J* = 8.4, 0.8 Hz, 1H), 6.80 (d, *J* = 8.4 Hz, 1H), 5.31 (s, 1H), and 3.92 (s, 3H). **4a** was demethylated directly with BBr_3_.

##### 2-(4-Hydroxyphenoxy)-5-methoxy-chromen-4-one (**4b**)

An amount of 80.0 mg (0.3 mmol) of **3a** was stirred with hydroquinone (109.0 mg, 1.0 mmol) and K_2_CO_3_ (273.7 mg, 2.0 mmol) in 1,4-dioxane (3.3 mL) at 80 °C. After 30 min, the reaction mixture was diluted with water (10.0 mL) and partitioned with ethyl acetate (10 mL × 3). The organic phases were combined and concentrated in vacuo. The crude product was purified by column chromatography (silica gel, dichloromethane: ethyl acetate = 2:5, *v*/*v*) to obtain **4b** as a brown solid (65.2 mg, yield: 69.5%). ^1^H NMR (400 MHz, CDCl_3_) δ 8.99 (s, 1H), 7.58 (t, *J* = 8.4, 8.4 Hz, 1H), 7.07 (dd, *J* = 8.8, 0.8 Hz, 1H), 6.94 (dd, *J* = 6.6, 2.4 Hz, 2H), 6.87 (d, *J* = 8.8 Hz, 1H), 6.84 (dd, *J* = 6.6, 2.4 Hz, 2H), 5.28 (s, 1H), and 3.97 (s, 3H). ^13^C NMR (100 MHz, CDCl_3_) δ 181.0 (C-4), 167.8 (C-2), 160.0 (C-5), 156.1 (C-9), 156.0 (C-4′), 143.5 (C-1′), 134.1 (C-7), 121.7 (C-2′, 6′), 116.8 (C-3′,5′), 112.9 (C-10), 109.8 (C-6), 107.5 (C-8), 90.0 (C-3), and 56.7 (5-OCH_3_). HRMS [ESI]^+^ calculated for [C_16_H_12_O_5_ + H]^+^ 285.0758; found [M + H]^+^ 285.0756.

##### 2-(4-Fluorophenoxy)-5-methoxy-chromen-4-one (**4c**)

An amount of 75.0 mg (0.3 mmol) of **3a** was stirred with 4-fluorophenol (103.1 mg, 0.9 mmol) and Cs_2_CO_3_ (599.5 mg, 1.8 mmol) in 1,4-dioxane (3.0 mL) at 80 °C. After 30 min, the reaction mixture was diluted with water (10.0 mL) and partitioned with ethyl acetate (10 mL × 3). The organic phases were combined and concentrated in vacuo. The crude product was purified by column chromatography (silica gel, hexane: ethyl acetate = 2:5, *v*/*v*) to obtain **4c** as a colorless gum (71.0 mg, yield: 80.0%). ^1^H NMR (400 MHz, CDCl_3_) δ 7.54 (t, *J* = 8.4, 8.4 Hz, 1H), 7.20–7.11 (m, 4H), 7.01 (dd, *J* = 8.4, 0.8 Hz, 1H), 6.85 (d, *J* = 8.4 Hz, 1H), 5.33 (s, 1H), and 3.97 (s, 3H). ^13^C NMR (100 MHz, CDCl_3_) δ 179.3, 166.0, 160.9 (d, *J* = 245.0 Hz), 160.0, 156.0, 147.5, 133.7, 122.7 (d, *J* = 9.0 Hz), 117.2 (d, *J* = 24.0 Hz), 113.4, 109.7, 107.5, 91.5, and 56.7. **4c** was demethylated directly with BBr_3_.

##### 5-Methoxy-2-(4-methoxyphenoxy)chromen-4-one (**4d**)

An amount of 70.0 mg (0.3 mmol) of **3a** was stirred with 4-methoxyphenol (106.8 mg, 0.9 mmol) and Cs_2_CO_3_ (280.2 mg, 0.9 mmol) in 1,4-dioxane (3.0 mL) at 80 °C. After 30 min, the reaction mixture was diluted with water (10.0 mL) and partitioned with ethyl acetate (10 mL × 3). The organic phases were combined and concentrated in vacuo. The crude product was purified by column chromatography (silica gel, dichloromethane: ethyl acetate = 3:2, *v*/*v*) to obtain **4d** as a white solid (86.4 mg, yield: 90.7%). ^1^H NMR (400 MHz, CDCl_3_) δ 7.53 (dd, *J* = 8.4, 8.0 Hz, 1H), 7.11 (d, *J* = 9.2 Hz, 2H), 7.01 (dd, *J* = 8.4, 0.8 Hz, 1H), 6.94 (d, *J* = 9.2 Hz, 2H), 6.83 (d, *J* = 8.0 Hz, 1H), 5.31 (s, 1H), 3.97 (s, 3H), and 3.83 (s, 3H). ^13^C NMR (100 MHz, CDCl_3_) δ 179.4, 166.6, 159.9, 158.1, 156.0, 144.9, 133.4, 122.0, 115.3, 113.4, 109.7, 107.3, 91.0, 56.6, and 55.8.

##### 5-Methoxy-2-(3,4,5-trimethoxyphenoxy)chromen-4-one (**4e**)

An amount of 70.0 mg (0.3 mmol) of **3a** was stirred with 4-methoxyphenol (158.4 mg, 0.9 mmol) and K_2_CO_3_ (118.9 mg, 0.9 mmol) in 1,4-dioxane (3.0 mL) at 80 °C. After 30 min, the reaction mixture was diluted with water (10.0 mL) and partitioned with ethyl acetate (10 mL × 3). The organic phases were combined and concentrated in vacuo. The crude product was purified by column chromatography (silica gel, dichloromethane: ethyl acetate = 2:1, *v*/*v*) to obtain **4e** as a white solid (77.4 mg, yield: 74.5%). ^1^H NMR (400 MHz, CDCl_3_) δ 7.54 (t, *J* = 8.4, 8.4 Hz, 1H), 7.02 (dd, *J* = 8.4, 0.8 Hz, 1H), 6.84 (dd, *J* = 8.4, 0.8Hz, 1H), 6.42 (s, 2H), 5.40 (s, 1H), 3.97 (s, 3H), 3.85 (s, 3H), and 3.84 (s, 6H). ^13^C NMR (100 MHz, CDCl_3_) δ 179.4 (C-4), 166.2 (C-2), 159.9 (C-5), 155.9 (C-9), 154.2 (C-3′,5′), 147.5 (C-1′), 136.6 (C-7), 133.6 (C-4′), 113.3 (C-10), 109.7 (C-6), 107.4 (C-8), 98.5 (C-8), 91.3 (C-2′,6′), 61.1 (4′-OCH_3_), 56.6(5-OCH_3_), and 56.4 (3′,5′-OCH_3_). HRMS [ESI]^+^ calculated for [C_19_H_18_O_7_ + H]^+^ 359.1125; found [M + H]^+^ 359.1127.

##### 6-Methoxy-2-phenoxy-chromen-4-one (**5a**)

An amount of 80.0 mg (0.3 mmol) of **3b** was stirred with phenol (92.6 mg, 1.0 mmol) and Cs_2_CO_3_ (534.3 mg, 1.6 mmol) in 1,4-dioxane (3.0 mL) at 80 °C. After 30 min, the reaction mixture was diluted with water (10.0 mL) and partitioned with ethyl acetate (10 mL × 3). The organic phases were combined and concentrated in vacuo. The crude product was purified by column chromatography (silica gel, hexane: ethyl acetate = 3:1, *v*/*v*) to obtain **5a** as a white solid (21.2 mg, yield: 24.1%). ^1^H NMR (400 MHz, CDCl_3_) δ 7.55 (d, *J* = 3.2 Hz, 1H), 7.49–7.43 (m, 2H), 7.38 (d, *J* = 8.8 Hz, 1H), 7.35–7.31 (m, 1H), 7.24 (d, *J* = 9.2, 3.2 Hz, 1H), 7.21–7.18 (m, 2H) 5.46 (s, 1H), and 3.89 (s, 3H). ^13^C NMR (100 MHz, CDCl_3_) δ 179.3 (C-4), 167.5 (C-2), 157.3 (C-6), 151.7 (C-1′), 148.4 (C-9), 130.5 (C-3′, 5′), 127.0 (C-7), 123.7 (C-10), 123.2 (C-4′), 121.0 (C-2′, 6′), 118.8 (C-8), 105.7 (C-5), 90.2 (C-3), and 56.1 (6-OCH_3_). HRMS [ESI]^+^ calculated for [C_16_H_12_O_4_ + H]^+^ 269.0808; found [M + H]^+^ 269.0807.

##### 2-(4-Hydroxyphenoxy)-6-methoxy-chromen-4-one (**5b**)

An amount of 80.0 mg (0.3 mmol) of **3b** was stirred with hydroquinone (109.0 mg, 1.0 mmol) and Cs_2_CO_3_ (136.8 mg, 1.0 mmol) in 1,4-dioxane (3.0 mL) at 80 °C. After 30 min, the reaction mixture was diluted with water (10.0 mL) and partitioned with ethyl acetate (10 mL × 3). The organic phases were combined and concentrated in vacuo. The crude product was purified by column chromatography (silica gel, chloroform: acetone = 13:1, *v*/*v*) to obtain **5b** as a brownish solid (65.7 mg, yield: 70.5%). ^1^H NMR (400 MHz, CD_3_OD) δ 7.52–7.49 (m, 2H), 7.38–7.34 (m, 1H), 7.11 (dd, *J* = 6.8, 2.4 Hz, 2H), 6.89 (dd, *J* = 6.8, 2.4 Hz, 2H), 5.35 (s, 1H), 4.59 (br, 1H), and 3.89 (s, 3H). ^13^C NMR (100 MHz, CD_3_OD) δ 181.5 (C-4), 170.3 (C-2), 159.0 (C-4′), 157.7 (C-6), 149.7 (C-9), 145.3 (C-1′), 124.3 (C-7), 124.2 (C-2′, 6′), 122.9 (C-10), 120.1 (C-8), 117.6 (C-3′, 5′), 106.6 (C-5), 89.7 (C-3), and 56.4 (6-OCH_3_). HRMS [ESI]^+^ calculated for [C_16_H_12_O_6_ + H]^+^ 285.0758; found [M + H]^+^ 285.0758.

##### 2-(4-Fluorophenoxy)-6-methoxy-chromen-4-one (**5c**)

An amount of 100.0 mg (0.4 mmol) of **3b** was stirred with 4-fluorophenol (137.9 mg, 1.2 mmol) and Cs_2_CO_3_ (801.5 mg, 2.5 mmol) in 1,4-dioxane (4.0 mL) at 80 °C. After 30 min, the reaction mixture was diluted with water (10.0 mL) and partitioned with ethyl acetate (10 mL × 3). The organic phases were combined and concentrated in vacuo. The crude product was purified by column chromatography (silica gel, hexane: ethyl acetate = 4:1, *v*/*v*) to obtain **5c** as a white solid (89.1 mg, yield: 75.9%). ^1^H NMR (400 MHz, CDCl_3_) δ 7.55 (d, *J* = 3.2 Hz, 1H), 7.37 (d, *J* = 9.2 Hz, 1H), 7.24 (dd, *J* = 9.2, 3.2 Hz, 1H), 7.21–7.12 (m, 4H), 5.44 (s, 1H), and 3.89 (s, 3H). ^13^C NMR (100 MHz, CDCl_3_) δ 179.2 (C-4), 167.4 (C-2), 160.9 (d, *J* = 245.0 Hz, C-4′), 157.4 (C-6), 148.3 (C-9), 147.5 (d, *J* = 2.0 Hz, C-1′), 123.7 (C-7), 123.2 (C-10), 122.6 (d, *J* = 9.0 Hz, C-2′, 6′), 118.8 (C-8), 117.2 (d, *J* = 24.0 Hz, C-3′, 5′), 105.7 (C-5), 90.0 (C-3), and 56.1 (6-OCH_3_). HRMS [ESI]^+^ calculated for [C_16_H_11_FO_4_ + H]^+^ 287.0714; found [M + H]^+^ 287.0712.

##### 6-Methoxy-2-(4-methoxyphenoxy)chromen-4-one (**5d**)

An amount of 80.0 mg (0.3 mmol) of **3b** was stirred with 4-methoxyphenol (124.1 mg, 1.0 mmol) and Cs_2_CO_3_ (136.8 mg, 1.0 mmol) in 1,4-dioxane (3.0 mL) at 80 °C. After 30 min, the reaction mixture was diluted with water (10.0 mL) and partitioned with ethyl acetate (10 mL × 3). The organic phases were combined and concentrated in vacuo. The crude product was purified by column chromatography (silica gel, hexane: ethyl acetate = 2:1, *v*/*v*) to obtain **5d** as a white solid (11.0 mg, yield: 11.2%). ^1^H NMR (400 MHz, CDCl_3_) δ 7.54 (d, *J* = 3.2 Hz, 1H), 7.37 (d, *J* = 8.8 Hz, 1H), 7.23 (dd, *J* = 8.8, 3.2 Hz, 1H), 7.12 (dd, *J* = 6.8, 2.4 Hz, 2H), 6.95 (dd, *J* = 6.8, 2.4 Hz, 2H), 5.42 (s, 1H), 3.88 (s, 3H), and 3.83 (s, 3H). ^13^C NMR (100 MHz, CDCl_3_) δ 179.3 (C-4), 168.2 (C-2), 158.2 (C-4′), 157.3 (C-6), 148.4 (C-9), 145.0 (C-1′), 123.7 (C-10), 123.1 (C-7), 122.0 (C-2′, 6′), 118.8 (C-8), 115.4 (C-3′, 5′), 105.7 (C-5), 89.7 (C-3), 56.1 (6-OCH_3_), and 55.9 (4′-OCH_3_). HRMS [ESI]^+^ calculated for [C_17_H_14_O_5_ + H]^+^ 299.0914; found [M + H]^+^ 299.0912.

##### 6-Methoxy-2-(3,4,5-trimethoxyphenoxy)chromen-4-one (**5e**)

An amount of 100.0 mg (0.4 mmol) of **3b** was stirred with 3,4,5-trimethoxyphenol (226.6 mg, 1.2 mmol) and Cs_2_CO_3_ (801.5 mg, 2.5 mmol) in 1,4-dioxane (3.0 mL) at 80 °C. After 30 min, the reaction mixture was diluted with water (10.0 mL) and partitioned with ethyl acetate (10 mL × 3). The organic phases were combined and concentrated in vacuo. The crude product was purified by column chromatography (silica gel, hexane: ethyl acetate = 5:2, *v*/*v*) to obtain **5e** as a brownish solid (43.6 mg, yield: 29.7%). ^1^H NMR (400 MHz, CDCl_3_) δ 7.55 (d, *J* = 2.8 Hz, 1H), 7.38 (d, *J* = 9.2 Hz, 1H), 7.24 (dd, *J* = 9.2, 2.8 Hz, 1H), 6.43 (s, 2H), 5.50 (s, 1H), 3.88 (s, 3H), 3.85 (s, 3H), and 3.84 (s, 6H). ^13^C NMR (100 MHz, CDCl_3_) δ 179.4 (C-4), 167.8 (C-2), 157.4 (C-6), 154.3 (C-3′, 5′), 148.4 (C-9), 147.5 (C-1′), 136.8 (C-4′), 123.7 (C-7), 123.2 (C-10), 118.8 (C-8), 105.7 (C-5), 98.6 (C-2′, 6′), 89.9 (C-3), 61.2 (4′-OCH_3_), 56.4 (3′, 5′-OCH_3_), and 56.1 (6-OCH_3_). HRMS [ESI]^+^ calculated for [C_19_H_18_O_7_ + H]^+^ 359.1125; found [M + H]^+^ 359.1127.

##### 7-Methoxy-2-phenoxy-chromen-4-one (**6a**)

An amount of 100.0 mg (0.4 mmol) of **3c** was stirred with phenol (115.6 mg, 1.2 mmol) and K_2_CO_3_ (113.3 mg, 0.8 mmol) in DMF (4.0 mL) at 80 °C. After 30 min, the reaction mixture was diluted with water (10.0 mL) and partitioned with ethyl acetate (10 mL × 3). The organic phases were combined and concentrated in vacuo. The crude product was purified by column chromatography (silica gel, hexane: ethyl acetate = 3:1, *v*/*v*) to obtain **6a** as a white solid (46.0 mg, yield: 36.0%). ^1^H NMR (400 MHz, CDCl_3_) δ 8.07 (d, *J* = 8.8 Hz, 1H), 7.48–7.44 (m, 2H), 7.35–7.31 (m, 1H), 7.22-7.18 (m, 2H), 6.98 (dd, *J* = 8.8, 2.4 Hz, 1H), 6.87 (d, *J* = 2.4 Hz, 1H), 5.39 (s, 1H), and 3.91 (s, 3H). ^13^C NMR (100 MHz, CDCl_3_) δ 178.7, 167.2, 163.9, 155.3, 151.5, 130.2, 127.0, 126.7, 120.7, 116.5, 114.0, 100.3, 90.1, and 55.8 [34].

##### 2-(4-Hydroxyphenoxy)-7-methoxy-chromen-4-one (**6b**)

An amount of 80.0 mg (0.3 mmol) of **3c** was stirred with hydroquinone (109.0 mg, 1.0 mmol) and K_2_CO_3_ (136.8 mg, 1.0 mmol) in DMF (3.0 mL) at 80 °C. After 30 min, the reaction mixture was diluted with water (10.0 mL) and partitioned with ethyl acetate (10 mL × 3). The organic phases were combined and concentrated in vacuo. The crude product was purified by column chromatography (silica gel, hexane: ethyl acetate = 1:1, *v*/*v*) to obtain **6b** as a brownish solid (75.2 mg, yield: 80.8%). ^1^H NMR (400 MHz, CDCl_3_) δ 8.41 (br, 1H), 8.10 (d, *J* = 8.8 Hz, 1H), 7.01 (dd, *J* = 8.8, 2.4 Hz, 1H), 6.98 (dd, *J* = 6.8, 2.4 Hz, 2H), 6.92 (d, *J* = 2.4 Hz, 1H), 6.83 (dd, *J* = 6.8, 2.4 Hz, 2H), 5.29 (s, 1H), and 3.93 (s, 3H). ^13^C NMR (100 MHz, Acetone-d6) δ 178.9 (C-4), 169.9 (C-2), 165.9(C-7), 157.8 (C-9), 157.2 (C-4′), 146.1(C-1′), 128.3 (C-5), 123.7 (C-2′, 6′), 118.3 (C-3′, 5′), 115.8 (C-10), 102.2 (C-8), 90.5 (C-3), and 57.4 (7-OCH_3_). HRMS [ESI]^+^ calculated for [C_16_H_12_O_5_ + H]^+^ 285.0758; found [M + H]^+^ 285.0758.

##### 2-(4-Fluorophenoxy)-7-methoxy-chromen-4-one (**6c**)

An amount of 60.0 mg (0.25 mmol) of **3c** was stirred with 4-fluorophenol (84.1 mg, 0.8 mmol) and Cs_2_CO_3_ (244.4 mg, 0.8 mmol) in 1,4-dioxane (2.5 mL) at 80 °C. After 30 min, the reaction mixture was diluted with water (10.0 mL) and partitioned with ethyl acetate (10 mL × 3). The organic phases were combined and concentrated in vacuo. The crude product was purified by column chromatography (silica gel, hexane: ethyl acetate = 3:1, *v*/*v*) to obtain **6c** as a brownish solid (66.3 mg, yield: 54.9%). ^1^H NMR (400 MHz, CDCl_3_) δ 8.06 (d, *J* = 8.8 Hz, 1H), 7.20–7.11 (m, 4H), 6.97 (dd, *J* = 8.8, 2.4 Hz, 1H), 6.84 (d, *J* = 2.4 Hz, 1H), 5.37 (s, 1H), and 3.90 (s, 3H). ^13^C NMR (100 MHz, CDCl_3_) δ 178.9 (C-4), 167.4 (C-2), 164.2 (C-7), 160.9 (d, *J* = 246.0 Hz, C-4′), 155.5 (C-9), 147.6 (d, *J* = 2.0 Hz, C-1′), 127.3 (C-5), 122.6 (d, *J* = 9.0 Hz, C-2′, 6′), 117.2 (d, *J* = 23.0 Hz, C-3′, 5′), 116.7 (C-10), 114.3 (C-6), 100.6 (C-8), 90.2 (C-3), and 56.0 (7-OCH_3_). HRMS [ESI]^+^ calculated for [C_16_H_11_FO_4_ + H]^+^ 287.0714; found [M + H]^+^ 287.0713.

##### 7-Methoxy-2-(4-methoxyphenoxy)chromen-4-one (**6d**)

An amount of 80.0 mg (0.3 mmol) of **3c** was stirred with 4-methoxyphenol (122.9 mg, 1.0 mmol) and K_2_CO_3_ (136.8 mg, 1.0 mmol) in DMF (3.0 mL) at 80 °C. After 30 min, the reaction mixture was diluted with water (10.0 mL) and partitioned with ethyl acetate (10 mL × 3). The organic phases were combined and concentrated in vacuo. The crude product was purified by column chromatography (silica gel, hexane: ethyl acetate = 2:1, *v*/*v*) to obtain **6d** as a yellowish solid (79.3 mg, yield: 81.0%). ^1^H NMR (400 MHz, CDCl_3_) δ 8.04 (d, *J* = 8.8 Hz, 1H), 7.11 (dd, *J* = 6.6, 2.0 Hz, 2H), 6.97–6.92 (m, 3H), 6.84 (d, *J* = 2.0 Hz, 1H), 5.34 (s, 1H), 3.89 (s, 3H), and 3.82 (s, 3H). ^13^C NMR (100 MHz, CDCl_3_) δ 178.8 (C-4), 167.9 (C-2), 163.9 (C-7), 158.0 (C-4′), 155.3 (C-9), 144.8 (C-1′), 127.0 (C-5), 121.8 (C-2′, 6′), 116.5 (C-10), 115.2 (C-3′,5′), 113.9 (C-6), 100.3 (C-8), 89.5 (C-2), 55.8 (OCH_3_), and 55.7 (OCH_3_).

##### 7-Methoxy-2-(3,4,5-trimethoxyphenoxy)chromen-4-one (**6e**)

An amount of 100.0 mg (0.4 mmol) of **3c** was stirred with 3,4,5-trimethoxyphenol (226.6 mg, 1.2 mmol) and K_2_CO_3_ (283.3 mg, 2.0 mmol) in DMF (4.0 mL) at 80 °C. After 30 min, the reaction mixture was diluted with water (10.0 mL) and partitioned with ethyl acetate (10 mL × 3). The organic phases were combined and concentrated in vacuo. The crude product was purified by column chromatography (silica gel, hexane: ethyl acetate = 1:1, *v*/*v*) to obtain 6e as a white solid (25.8 mg, yield: 17.6%). ^1^H NMR (400 MHz, CDCl_3_) δ 8.07 (d, *J* = 8.8 Hz, 1H), 6.98 (dd, *J* = 8.8, 2.4 Hz, 1H), 6.87 (d, *J* = 2.4 Hz, 1H), 6.43 (s, 2H), 5.44 (s, 1H), 3.91 (s, 3H), 3.86 (s, 3H), and 3.85 (s, 6H). ^13^C NMR (100 MHz, CDCl_3_) δ 179.0 (C-4), 167.7 (C-2), 164.2 (C-7), 155.5 (C-9), 154.3 (C-3′, 5′), 147.6 (C-1′), 136.7 (C-4′), 127.3 (C-5), 116.7 (C-10), 114.2 (C-6), 100.6 (C-8), 98.6 (C-2′, 6′), 90.0 (C-3), 61.2 (4′-OCH_3_), 56.4 (3′, 5′-OCH_3_), and 56.0 (7-OCH_3_). HRMS [ESI]^+^ calculated for [C_19_H_18_O_7_ + H]^+^ 359.1125; found [M + H]^+^ 359.1123.

##### 5,7-Dimethoxy-2-phenoxy-chromen-4-one (**7a**)

An amount of 60.0 mg (0.2 mmol) of **3d** was stirred with hydroquinone (62.1 mg, 0.7 mmol) and Cs_2_CO_3_ (430.1 mg, 1.3 mmol) in 1,4-dioxane (2.0 mL) at 80 °C. After 30 min, the reaction mixture was diluted with water (10.0 mL) and partitioned with ethyl acetate (10 mL × 3). The organic phases were combined and concentrated in vacuo. The crude product was purified by column chromatography (silica gel, dichloromethane: ethyl acetate = 3:2, *v*/*v*) to obtain **7a** as a white solid (64.2 mg, yield: 97.8%). ^1^H NMR (400 MHz, CDCl_3_) δ 7.44 (t, *J* = 8.0, 8.0 Hz, 2H), 7.31 (t, *J* = 8.0, 8.0 Hz, 1H), 7.18 (d, *J* = 8.0 Hz, 2H), 6.47 (d, *J* = 2.0 Hz, 1H), 6.38 (d, *J* = 2.0 Hz, 1H), 5.29 (s, 1H), 3.93 (s, 3H), and 3.88 (s, 3H). ^13^C NMR (100 MHz, CDCl_3_) δ 178.9 (C-4), 165.7 (C-2), 163.9 (C-7), 161.0 (C-5), 157.6 (C-9), 151.8 (C-1′), 130.4 (C-3′, 5′), 126.8 (C-4′), 120.9 (C-2′, 6′), 107.9 (C-10), 96.6 (C-6), 92.9 (C-8), 91.4 (C-3), 56.6 (OCH_3_), and 55.9 (OCH_3_). HRMS [ESI]^+^ calculated for [C_17_H_14_O_5_ + H]^+^ 299.0914; found [M + H]^+^ 299.0912.

##### 2-(4-Hydroxyphenoxy)-5,7-dimethoxy-chromen-4-one (**7b**)

An amount of 95.4 mg (0.35 mmol) of **3d** was stirred with hydroquinone (120.0 mg, 1.1 mmol) and K_2_CO_3_ (387.0 mg, 2.8 mmol) in 1,4-dioxane (3.5 mL) at 80 °C. After 30 min, the reaction mixture was diluted with water (10.0 mL) and partitioned with ethyl acetate (10 mL × 3). The organic phases were combined and concentrated in vacuo. The crude product was purified by column chromatography (silica gel, dichloromethane: ethyl acetate = 1:2, *v*/*v*) to obtain **7b** as a white solid (20.1 mg, yield: 18.3%). ^1^H NMR (400 MHz, CD_3_OD) δ 7.09 (dd, *J* = 6.6, 2.0 Hz, 2H), 6.88 (dd, *J* = 6.6, 2.0 Hz, 2H), 6.64 (d, *J* = 2.0 Hz, 1H), 6.54 (d, *J* = 2.0 Hz, 1H), 5.14 (s, 1H), 4.59 (br, 1H), 3.91 (s, 3H), and 3.88 (s, 3H). ^13^C NMR (100 MHz, CD_3_OD) δ 181.5, 168.7, 166.3, 162.1, 159.0, 157.6, 145.4, 122.8, 117.6, 107.8, 97.6, 94.2, 90.5, and 56.6 [17].

##### 2-(4-Fluorophenoxy)-5,7-dimethoxy-chromen-4-one (**7c**)

An amount of 80.0 mg (0.3 mmol) of **3d** was stirred with hydroquinone (100.9 mg, 0.9 mmol) and Cs_2_CO_3_ (586.5 mg, 1.8 mmol) in 1,4-dioxane (3.0 mL) at 80 °C. After 30 min, the reaction mixture was diluted with water (10.0 mL) and partitioned with ethyl acetate (10 mL × 3). The organic phases were combined and concentrated in vacuo. The crude product was purified by column chromatography (silica gel, dichloromethane: ethyl acetate = 2:1, *v*/*v*) to obtain **7c** as a white solid (80.7 mg, yield: 85.1%). ^1^H NMR (400 MHz, CDCl_3_) δ 7.19–7.10 (m, 4H), 6.45 (d, *J* = 2.4 Hz, 1H), 6.38 (d, *J* = 2.4 Hz, 1H), 5.26 (s, 1H), 3.93 (s, 3H), and 3.88 (s, 3H). ^13^C NMR (100 MHz, CDCl_3_) δ 178.7 (C-4), 165.7 (C-2), 163.9 (C-7), 161.0 (C-5), 160.7 (d, *J* = 245.0 Hz, C-4′), 157.6 (C-9), 147.6 (d, *J* = 3.0 Hz, C-1′), 122.5 (d, *J* = 9.0 Hz, C-2′, 6′), 117.1 (d, *J* = 24.0 Hz, C-3′, 5′), 107.8 (C-10), 96.6 (C-7), 92.9 (C-8), 91.2 (C-3), 56.6 (OCH_3_), and 55.9 (OCH_3_). HRMS [ESI]^+^ calculated for [C_17_H_13_FO_5_ + H]^+^ 317.0820; found [M + H]^+^ 317.0817.

##### 5,7-Dimethoxy-2-(4-methoxyphenoxy)chromen-4-one (**7d**)

An amount of 250.0 mg (0.9 mmol) of **3d** was stirred with 4-methoxyphenol (335.2 mg, 2.7 mmol) and Cs_2_CO_3_ (879.7 mg, 2.7 mmol) in 1,4-dioxane (9.0 mL) at 80 °C. After 30 min, the reaction mixture was diluted with water (20.0 mL) and partitioned with ethyl acetate (20 mL × 3). The organic phases were combined and concentrated in vacuo. The crude product was purified by column chromatography (silica gel, dichloromethane: ethyl acetate = 1:1, *v*/*v*) to obtain **7d** as a white solid (271.6 mg, yield: 90.9%). ^1^H NMR (400 MHz, CDCl_3_) δ 7.10 (dd, *J* = 6.8, 2.0 Hz, 1H), 6.93 (dd, *J* = 6.8, 2.4 Hz, 1H), 6.46 (d, *J* = 2.0 Hz, 1H), 6.37 (d, *J* = 2.4 Hz, 1H), 5.25 (s, 1H), 3.92 (s, 3H), 3.88 (s, 3H), and 3.82 (s, 3H). ^13^C NMR (100 MHz, CDCl_3_) δ 178.9, 166.4, 163.9, 161.0, 158.1, 157.6, 145.1, 122.0, 115.4, 107.9, 96.6, 92.9, 90.8, 56.6, and 55.9 [35].

##### 5,7-Dimethoxy-2-(3,4,5-trimethoxyphenoxy)chromen-4-one (**7e**)

An amount of 70.0 mg (0.26 mmol) of **3d** was stirred with 3,4,5-trimethoxyphenol (143.7 mg, 0.8 mmol) and Cs_2_CO_3_ (254.1 mg, 0.8 mmol) in 1,4-dioxane (2.6 mL) at 80 °C. After 30 min, the reaction mixture was diluted with water (10.0 mL) and partitioned with ethyl acetate (10 mL × 3). The organic phases were combined and concentrated in vacuo. The crude product was purified by column chromatography (silica gel, chloroform: ethyl acetate = 2:3 = 3:2, *v*/*v*) to obtain **7e** as a brownish solid (82.0 mg, yield: 81.2%). ^1^H NMR (400 MHz, CDCl_3_) δ 5.47 (d, *J* = 2.4 Hz, 1H), 5.41 (s, 2H), 5.39 (d, *J* = 2.4 Hz, 1H), 5.34 (s, 1H), 3.93 (s, 3H), 3.89 (s, 3H), 3.85 (s, 3H), and 3.83 (s, 6H). ^13^C NMR (100 MHz, CDCl_3_) δ 178.7 (C-4), 165.8 (C-2), 163.7 (C-7), 160.8 (C-5), 157.4 (C-9), 154.0 (C-3′, 5′), 147.5 (C-1′), 136.4 (C-4′), 107.6 (C-10), 98.3 (C-2′, 6′), 96.4 (C-6), 92.7 (C-8), 90.9 (C-3), 60.9 (4′-OCH_3_), 56.3 (OCH_3_), 56.2 (3′, 5′-OCH_3_), and 55.7 (OCH_3_). HRMS [ESI]^+^ calculated for [C_20_H_20_O_8_ + H]^+^ 389.1231; found [M + H]^+^ 389.1232.

##### 5,6,7-Trimethoxy-2-phenoxy-chromen-4-one (**8a**)

An amount of 100.0 mg (0.3 mmol) of **3e** was stirred with phenol (93.2 mg, 1.0 mmol) and Cs_2_CO_3_ (645.1 mg, 1.9 mmol) in 1,4-dioxane (3.0 mL) at 80 °C. After 30 min, the reaction mixture was diluted with water (10.0 mL) and partitioned with ethyl acetate (10 mL × 3). The organic phases were combined and concentrated in vacuo. The crude product was purified by column chromatography (silica gel, hexane: ethyl acetate = 3:2, *v*/*v*) to obtain **8a** as a white solid (90.6 mg, yield: 88.6%). ^1^H NMR (400 MHz, CDCl_3_) δ 7.46–7.15 (m, 5H), 6.68 (s, 1H), 5.29 (s, 1H), 3.93 (s, 6H), and 3.88 (s, 3H). ^13^C NMR (100 MHz, CDCl_3_) δ 178.4 (C-4), 165.9 (C-2), 157.5 (C-7), 152.9 (C-9), 152.0 (C-5), 151.8 (C-1′), 140.8 (C-6), 130.4 (C-3′, 5′), 126.8 (C-4′), 120.8 (C-2′, 6′), 111.6 (C-10), 96.2 (C-8), 91.1 (C-3), 62.3 (5-OCH_3_), 61.6 (6-OCH_3_), and 56.4 (7-OCH_3_). HRMS [ESI]^+^ calculated for [C_18_H_16_O_6_ + H]^+^ 329.1020; found [M + H]^+^ 329.1021.

##### 2-(4-Hydroxyphenoxy)-5,6,7-trimethoxy-chromen-4-one (**8b**)

An amount of 80.0 mg (0.26 mmol) of **3e** was stirred with hydroquinone (88.9 mg, 0.8 mmol) and K_2_CO_3_ (215.6 mg, 1.6 mmol) in 1,4-dioxane (2.6 mL) at 80 °C. After 30 min, the reaction mixture was diluted with water (10.0 mL) and partitioned with ethyl acetate (10 mL × 3). The organic phases were combined and concentrated in vacuo. The crude product was purified by column chromatography (silica gel, hexane: ethyl acetate = 2:3, *v*/*v*) to obtain **8b** as a brownish solid (88.1 mg, yield: 98.4%). ^1^H NMR (400 MHz, CDCl_3_) δ 9.45 (s, 1H), 6.92 (dd, *J* = 6.8, 2.4 Hz, 2H), 6.81 (dd, *J* = 6.8, 2.4 Hz, 2H), 6.77 (s, 1H), 5.19 (s, 1H), 3.97 (s, 3H), 3.96 (s, 3H), and 3.91 (s, 3H). ^13^C NMR (100 MHz, CDCl_3_) δ 180.2 (C-4), 168.1 (C-2), 158.1 (C-7), 156.2 (C-4′), 152.9 (C-9), 152.2 (C-5), 143.4 (C-1′), 141.1 (C-6), 121.7 (C-2′, 6′), 116.7 (C-3′, 5′), 111.1 (C-10), 96.3 (C-8), 89.0 (C-3), 62.5 (5-OCH_3_), 61.7 (6-OCH_3_), and 56.6 (7-OCH_3_). HRMS [ESI]^+^ calculated for [C_18_H_16_O_7_ + H]^+^ 345.0969; found [M + H]^+^ 345.0970.

##### 2-(4-Fluorophenoxy)-5,6,7-trimethoxy-chromen-4-one (**8c**)

An amount of 100.0 mg (0.3 mmol) of **3e** was stirred with 4-fluorophenol (111.0 mg, 1.0 mmol) and Cs_2_CO_3_ (645.1 mg, 1.9 mmol) in 1,4-dioxane (3.0 mL) at 80 °C. After 30 min, the reaction mixture was diluted with water (10.0 mL) and partitioned with ethyl acetate (10 mL × 3). The organic phases were combined and concentrated in vacuo. The crude product was purified by column chromatography (silica gel, hexane: ethyl acetate = 3:2, *v*/*v*) to obtain **8c** as a yellowish solid (99.5 mg, yield: 87.1%). ^1^H NMR (400 MHz, CDCl_3_) δ 7.19–7.11 (m, 4H), 6.81 (s, 1H), 5.28 (s, 1H), 3.94 (s, 3H), 3.94 (s, 3H), and 3.90 (s, 3H). ^13^C NMR (100 MHz, CDCl_3_) δ 178.3 (C-4), 165.9 (C-2), 160.8 (d, *J* = 246.0 Hz, C-4′), 157.6 (C-7), 152.9 (C-9), 152.0 (C-5), 147.6 (d, *J* = 3.0 Hz, C-1′), 140.8 (C-6) 122.5 (d, *J* = 9.0 Hz, C-2′, 6′), 117.2 (d, *J* = 24.0 Hz, C-3′, 5′), 111.6 (C-10), 96.1 (C-8), 90.9 (C-3), 62.3 (5-OCH_3_), 61.6 (6-OCH_3_), and 56.4 (7-OCH_3_). HRMS [ESI]^+^ calculated for [C_18_H_15_FO_6_ + H]^+^ 347.0925; found [M + H]^+^ 347.0926.

##### 5,6,7-Trimethoxy-2-(4-methoxyphenoxy)chromen-4-one (**8d**)

An amount of 80.0 mg (0.26 mmol) of **3e** was stirred with 4-methoxyphenol (96.8 mg, 0.8 mmol) and Cs_2_CO_3_ (215.6 mg, 0.8 mmol) in 1,4-dioxane (2.6 mL) at 80 °C. After 30 min, the reaction mixture was diluted with water (10.0 mL) and partitioned with ethyl acetate (10 mL × 3). The organic phases were combined and concentrated in vacuo. The crude product was purified by column chromatography (silica gel, hexane: ethyl acetate = 3:2, *v*/*v*) to obtain **8d** as a white solid (75.8 mg, yield: 81.4%). ^1^H NMR (400 MHz, CDCl_3_) δ 7.10 (dd, *J* = 6.8, 2.4 Hz, 2H), 6.94 (dd, *J* = 6.8, 2.4 Hz, 2H), 6.69 (s, 1H), 5.26 (s, 1H), 3.94 (s, 3H), 3.93 (s, 3H), 3.89 (s, 3H), and 3.83 (s, 3H). ^13^C NMR (100 MHz, CDCl_3_) δ 178.4, 166.6, 158.1, 157.4, 152.9, 152.0, 145.1, 140.7, 121.9, 115.3, 111.6, 96.1, 90.5, 62.3, 61.6, 56.4, and 55.8.

##### 5,6,7-Trimethoxy-2-(3,4,5-trimethoxyphenoxy)chromen-4-one (**8e**)

An amount of 60.0 mg (0.2 mmol) of **3e** was stirred with 4-methoxyphenol (110.5 mg, 0.6 mmol) and Cs_2_CO_3_ (391.0 mg, 1.2 mmol) in 1,4-dioxane (2.5 mL) at 80 °C. After 30 min, the reaction mixture was diluted with water (10.0 mL) and partitioned with ethyl acetate (10 mL × 3). The organic phases were combined and concentrated in vacuo. The crude product was purified by column chromatography (silica gel, dichloromethane: ethyl acetate = 5:1, *v*/*v*) to obtain **8e** as a brownish solid (71.3 mg, yield: 85.2%). ^1^H NMR (400 MHz, CDCl_3_) δ 6.69 (s, 1H), 6.41 (s, 2H), 5.34 (s, 1H), 3.94 (s, 6H), 3.89 (s, 3H), 3.85 (s, 3H), and 3.84 (s, 6H). ^13^C NMR (100 MHz, CDCl_3_) δ 178.4 (C-4), 166.2 (C-2), 157.5 (C-7), 154.2 (C-3′, 5′), 152.9 (C-9), 152.0 (C-5), 147.6 (C-1′), 140.8 (C-6), 136.7 (C-4′), 111.6 (C-10), 98.5 (C-2′, 6′), 96.1 (C-8), 90.8 (C-3), 62.3 (OCH_3_), 61.6 (5-OCH_3_), 61.1 (4′-OCH_3_), and 56.4 (7, 3′, 5′-OCH_3_). HRMS [ESI]^+^ calculated for [C_21_H_22_O_9_ + H]^+^ 419.1337; found [M + H]^+^ 419.1336.

##### 7-Fluoro-2-phenoxy-chromen-4-one (**9a**)

An amount of 100.0 mg (0.4 mmol) of **3f** was stirred with phenol (115.8 mg, 1.2 mmol) and Cs_2_CO_3_ (801.5 mg, 2.5 mmol) in 1,4-dioxane (4.0 mL) at 80 °C. After 30 min, the reaction mixture was diluted with water (10.0 mL) and partitioned with ethyl acetate (10 mL × 3). The organic phases were combined and concentrated in vacuo. The crude product was purified by column chromatography (silica gel, hexane: ethyl acetate = 5:1, *v*/*v*) to obtain **9a** as a white solid (91.2 mg, yield: 86.8%). ^1^H NMR (400 MHz, CDCl_3_) δ 8.17 (dd, *J* = 9.6, 6.4 Hz, 1H), 7.49–7.44 (m, 2H), 7.37–7.32 (m, 1H), 7.22–7.11 (m, 4H), and 5.42 (s, 1H). ^13^C NMR (100 MHz, CDCl_3_) δ 178.4 (C-4), 167.9 (C-2), 165.6 (d, *J* = 254.0 Hz, C-7), 154.7 (d, *J* = 13.0 Hz, C-9), 151.5 (C-1′), 130.6 (C-3′, 5′), 128.3 (d, *J* = 10.0 Hz, C-5), 127.2 (C-4′), 120.9 (C-2′,6′), 119.9 (C-10), 114.2 (d, *J* = 23.0 Hz, C-6), 104.6 (d, *J* = 25.0 Hz, C-8), and 90.5 (C-3). HRMS [ESI]^+^ calculated for [C_15_H_9_FO_3_ + H]^+^ 257.0608; found [M + H]^+^ 257.0610.

##### 7-Fluoro-2-(4-hydroxyphenoxy)chromen-4-on (**9b**)

An amount of 100.0 mg (0.4 mmol) of **3f** was stirred with hydroquinone (135.4 mg, 1.2 mmol) and K_2_CO_3_ (340.0 mg, 2.5 mmol) in 1,4-dioxane (4.0 mL) at 80 °C. After 30 min, the reaction mixture was diluted with water (10.0 mL) and partitioned with ethyl acetate (10 mL × 3). The organic phases were combined and concentrated in vacuo. The crude product was purified by column chromatography (silica gel, hexane: ethyl acetate = 3:2, *v*/*v*) to obtain **9b** as a brown solid (89.8 mg, yield: 80.5%). ^1^H NMR (400 MHz, Acetone-d6) δ 8.72 (br, 1H), 8.11–8.08 (m, 1H), 7.42–7.38 (m, 1H), 7.42–7.31 (m, 1H), 7.21 (dd, *J* = 6.8, 2.4 Hz, 2H), 6.98 (dd, *J* = 6.8, 2.4 Hz, 2H), and 5.21 (s, 1H). ^13^C NMR (100 MHz, Acetone-d6) δ 177.6 (C-4), 169.4 (C-2), 166.3 (d, *J* = 251.0 Hz, C-7), 157.1 (C-4′), 155.6 (d, *J* = 13.0 Hz, C-9), 145.1 (C-1′), 128.7 (d, *J* = 11.0 Hz, C-5), 122.8 (C-2′,6′), 120.9 (C-10), 117.5(C-3′,5′), 114.6 (d, *J* = 23.0 Hz, C-6), 105.4 (d, *J* = 26.0 Hz, C-8), and 89.9 (C-3). HRMS [ESI]^+^ calculated for [C_15_H_9_FO_4_ + H]^+^ 273.0558; found [M + H]^+^ 273.0558.

##### 7-Fluoro-2-(4-fluorophenoxy)chromen-4-one (**9c**)

An amount of 100.0 mg (0.4 mmol) of **3f** was stirred with 4-fluorophenol (137.9 mg, 1.2 mmol) and Cs_2_CO_3_ (801.5 mg, 2.5 mmol) in 1,4-dioxane (4.0 mL) at 80 °C. After 30 min, the reaction mixture was diluted with water (10.0 mL) and partitioned with ethyl acetate (10 mL × 3). The organic phases were combined and concentrated in vacuo. The crude product was purified by column chromatography (silica gel, hexane: ethyl acetate = 20:3, *v*/*v*) to obtain **9c** as a white solid (52.0 mg, yield: 46.2%). ^1^H NMR (400 MHz, CDCl_3_) δ 8.17 (dd, *J* = 9.2, 6.4 Hz, 1H), 7.21–7.13 (m, 6H), and 5.41 (s, 1H). ^13^C NMR (100 MHz, CDCl_3_) δ 178.2 (C-4), 167.8 (C-2), 165.6 (d, *J* = 253.0 Hz, C-7), 161.0 (d, *J* = 246.0 Hz, C-4′), 154.7 (d, *J* = 14.0 Hz, C-9), 147.3 (d, *J* = 3.0 Hz, C-1′), 128.3 (d, *J* = 10.0 Hz, C-5), 122.6 (d, *J* = 9.0 Hz, C-2′,4′), 119.8 (C-10), 117.3 (d, *J* = 24.0 Hz, C-3′, 5′), 114.3 (d, *J* = 23.0 Hz, C-6), 104.6 (d, *J* = 26.0 Hz, C-8), and 90.3 (C-3). HRMS [ESI]^+^ calculated for [C_15_H_8_F_2_O_3_ + H]^+^ 275.0514; found [M + H]^+^ 275.0513.

##### 7-Fluoro-2-(4-methoxyphenoxy)chromen-4-one (**9d**)

An amount of 70.0 mg (0.3 mmol) of **3f** was stirred with 4-methoxyphenol (105.5 mg, 0.9 mmol) and K_2_CO_3_ (279.5 mg, 0.9 mmol) in 1,4-dioxane (4.0 mL) at 80 °C. After 30 min, the reaction mixture was diluted with water (10.0 mL) and partitioned with ethyl acetate (10 mL × 3). The organic phases were combined and concentrated in vacuo. The crude product was purified by column chromatography (silica gel, hexane: ethyl acetate = 10:3, *v*/*v*) to obtain **9d** as a white solid (76.6 mg, yield: 95.6%). ^1^H NMR (400 MHz, CDCl_3_) δ 8.17 (dd, *J* = 9.2, 6.4 Hz, 1H), 7.16–7.10 (m, 4H), 6.96 (dd, *J* = 7.0, 2.4 Hz, 2H), 5.40 (s, 1H), and 3.84 (s, 3H). ^13^C NMR (100 MHz, CDCl_3_) δ 178.3 (C-4), 168.4 (C-2), 165.6 (d, *J* = 253.0 Hz, C-7), 158.3 (C-4′), 154.7 (d, *J* = 13.0 Hz, C-9), 144.8 (C-1′), 128.3 (d, *J* = 10.0 Hz, C-5), 121.9 (C-2′, 6′), 119.9 (C-10), 115.4 (C-3′, 5′), 114.1 (d, *J* = 23.0 Hz, C-6), 104.6 (d, *J* = 26.0 Hz, C-8), 89.9 (C-3), and 55.9 (4′-OCH_3_). HRMS [ESI]^+^ calculated for [C_16_H_11_FO_4_ + H]^+^ 287.0714; found [M + H]^+^ 287.0713.

##### 7-Fluoro-2-(3,4,5-trimethoxyphenoxy)chromen-4-one (**9e**)

An amount of 90.0 mg (0.4 mmol) of **3f** was stirred with 3,4,5-trimethoxyphenol (204.5 mg, 1.1 mmol) and Cs_2_CO_3_ (723.3 mg, 2.2 mmol) in 1,4-dioxane (4.0 mL) at 80 °C. After 30 min, the reaction mixture was diluted with water (10.0 mL) and partitioned with ethyl acetate (10 mL × 3). The organic phases were combined and concentrated in vacuo. The crude product was purified by column chromatography (silica gel, chloroform: acetone = 15:1, *v*/*v*) to obtain **9e** as a brownish solid (86.6 mg, yield: 67.9%). ^1^H NMR (400 MHz, CDCl_3_) δ 8.17 (ddd, *J* = 6.6, 6.6, 1.2 Hz, 1H), 7.18–7.13 (m, 2H), 6.43 (s, 2H), 5.48 (s, 1H), 3.86 (s, 3H), and 3.85 (s, 6H). ^13^C NMR (100 MHz, CDCl_3_) δ 178.4 (C-4), 168.1 (C-2), 165.7 (d, *J* = 254.0 Hz, C-7), 154.7 (d, *J* = 13.0 Hz, C-9), 154.4 (C-3′, 5′), 147.4 (C-1′), 136.9 (C-4′), 128.3 (d, *J* = 11.0 Hz, C-5), 119.9 (C-10), 114.2 (d, *J* = 23.0 Hz, C-6), 104.6 (d, *J* = 26.0 Hz, C-8), 98.5 (C-2′, 6′), 90.2 (C-3), 61.2 (4′-OCH_3_), and 56.5 (3′, 5′-OCH_3_). HRMS [ESI]^+^ calculated for [C_18_H_15_FO_6_ + H]^+^ 347.0925; found [M + H]^+^ 347.0927.

##### 5-Hydroxy-2-(4-hydroxyphenoxy)chromen-4-one (**10a**)

An amount of 85.0 mg (0.3 mmol) of **4b** was dissolved in anhydrous DCM (1.2 mL) and then treated with BBr_3_ (1 M in DCM, 0.9 mL) dropwise at 0 °C in an inert atmosphere. After 1 h had passed, the resulting solution was continuously stirred at ambient temperature overnight, and the reaction mixture was diluted with water (10 mL) and partitioned with ethyl acetate (10 mL × 3). The organic phases were combined and concentrated in vacuo. The crude product was purified by column chromatography (silica gel, chloroform: acetone = 10:1, *v*/*v*) to produce **10a** as a white solid (44.1 mg, yield: 54.4%). ^1^H NMR (400 MHz, Acetone-d6) δ 12.78 (s, 1H), 8.74 (s, 1H), 7.61 (t, *J* = 8.8, 8.8 Hz, 1H), 7.23 (dd, *J* = 6.8, 2.4 Hz, 2H), 6.99 (dd, *J* = 6.8, 2.4 Hz, 2H), 6.96 (dd, *J* = 8.8, 0.8 Hz, 1H), 6.78 (dd, *J* = 8.8, 0.8 Hz, 1H), and 5.22 (s, 1H). ^13^C NMR (100 MHz, Acetone-d6) δ 185.5, 169.9, 161.9, 157.3, 154.9, 144.9, 136.0, 122.9, 117.6, 112.7, 109.8, 107.4, and 88.7.

##### 2-(4-Fluorophenoxy)-5-hydroxy-chromen-4-one (**10b**)

An amount of 70.0 mg (0.2 mmol) of **4c** was dissolved in anhydrous DCM (0.8 mL) and then treated with BBr_3_ (1 M in DCM, 0.6 mL) dropwise at 0 °C in an inert atmosphere. After 1 h had passed, the resulting solution was continuously stirred at ambient temperature. After 3.5 h, the reaction mixture was diluted with water (10 mL) and partitioned with ethyl acetate (10 mL × 3). The organic phases were combined and concentrated in vacuo. The crude product was purified by column chromatography (silica gel, chloroform) to produce **10b** as a white solid (58.2 mg, yield: 93.0%). ^1^H NMR (400 MHz, CDCl_3_) δ 12.53 (s, 1H), 7.50 (t, *J* = 8.4, 8.4 Hz, 1H), 7.20–7.15 (m, 4H), 6.87 (d, *J* = 8.4 Hz, 1H), 6.82 (d, *J* = 8.4 Hz, 1H), and 5.34 (s, 1H). ^13^C NMR (100 MHz, CDCl_3_) δ 184.8, 167.9, 161.0 (d, *J* = 245.0 Hz), 159.8, 153.9, 147.1 (d, *J* = 3.0 Hz), 135.0, 122.6 (d, *J* = 9.0 Hz), 117.4 (d, *J* = 24.0 Hz), 112.5, 109.3, 106.6, and 88.9.

##### 6-Hydroxy-2-(4-hydroxyphenoxy)chromen-4-one (**11a**)

An amount of 105.0 mg (0.4 mmol) of **5b** was dissolved in anhydrous DCM (1.5 mL) and then treated with BBr_3_ (1 M in DCM, 1.2 mL) dropwise at 0 °C in an inert atmosphere. After 1 h had passed, the resulting solution was continuously stirred at ambient temperature overnight, and the reaction mixture was diluted with water (10 mL) and partitioned with ethyl acetate (10 mL × 3). The organic phases were combined and concentrated in vacuo. The crude product was purified by column chromatography (silica gel, dichloromethane: ethyl acetate = 3:2, *v*/*v*) to produce **11a** as a yellowish solid (85.3 mg, yield: 85.3%). ^1^H NMR (400 MHz, CD_3_OD) δ 7.42 (d, *J* = 9.2 Hz, 1H), 7.37 (d, *J* = 2.8 Hz, 1H), 7.22 (dd, *J* = 9.2, 2.8 Hz, 1H), 7.10 (dd, *J* = 6.8, 2.0 Hz, 2H), 6.89 (dd, *J* = 6.8, 2.0 Hz, 2H), and 5.31 (s, 1H). ^13^C NMR (100 MHz, CD_3_OD) δ 181.7 (C-4), 170.3 (C-2), 157.6 (C-4′), 156.8 (C-6), 148.9 (C-9), 145.3 (C-1′), 124.4 (C-10), 123.9 (C-7), 122.9 (C-2′, 6′), 119.8 (C-8), 117.6 (C-3′, 5′), 109.5 (C-5), and 89.5 (C-3). HRMS [ESI]^+^ calculated for [C_15_H_10_O_5_ + H]^+^ 271.0601; found [M + H]^+^ 271.0600.

##### 2-(4-Fluorophenoxy)-6-hydroxy-chromen-4-one (**11b**)

An amount of 83.9 mg (0.3 mmol) of **5c** was dissolved in anhydrous DCM (1.0 mL) and then treated with BBr_3_ (1 M in DCM, 0.9 mL) dropwise at 0 °C in an inert atmosphere. After 1 h had passed, the resulting solution was continuously stirred at ambient temperature. After 4 h, the reaction mixture was diluted with water (10 mL) and partitioned with ethyl acetate (10 mL × 3). The organic phases were combined and concentrated in vacuo. The crude product was purified by column chromatography (silica gel, dichloromethane: ethyl acetate = 4:1, *v*/*v*) to produce **11b** as an orangish solid (60.0 mg, yield: 73.5%). ^1^H NMR (400 MHz, CD_3_OD) δ 7.92 (d, *J* = 8.4 Hz, 1H), 7.37–7.32 (m, 2H), 7.33–7.24 (m, 2H), 6.93 (dd, *J* = 8.4, 2.4 Hz, 1H), 6.83 (d, *J* = 2.4 Hz, 1H), and 5.29 (s, 1H). ^13^C NMR (100 MHz, CD_3_OD) δ 181.3 (C-4), 169.3 (C-2), 164.9 (C-6), 162.3 (d, *J* = 243.0 Hz, C-4′), 157.2 (C-9), 149.0 (C-1′), 127.9 (C-7), 123.9 (d, *J* = 9.0 Hz, C-2′, 6′), 118.1 (d, *J* = 24.0 Hz, C-3′, 5′), 116.3 (C-10), 116.0 (C-8), 103.3 (C-5), and 90.0 (C-3). HRMS [ESI]^+^ calculated for [C_15_H_9_FO_4_ + H]^+^ 273.0558; found [M + H]^+^ 273.0556.

##### 7-Hydroxy-2-(4-hydroxyphenoxy)chromen-4-one (**12a**)

An amount of 80.0 mg (0.3 mmol) of **6b** was dissolved in anhydrous DCM (1.0 mL) and then treated with BBr_3_ (1 M in DCM, 0.9 mL) dropwise at 0 °C in an inert atmosphere. After 1 h had passed, the resulting solution was continuously stirred at ambient temperature overnight, and the reaction mixture was diluted with water (10 mL) and partitioned with ethyl acetate (10 mL × 3). The organic phases were combined and concentrated in vacuo. The crude product was purified by column chromatography (silica gel, hexane: ethyl acetate = 1:2, *v*/*v*) to produce **12a** as an orangish solid (65.3 mg, yield: 86.3%). ^1^H NMR (400 MHz, Acetone-d6) δ 7.90 (d, *J* = 8.4 Hz, 1H), 7.19 (dd, *J* = 6.4, 2.4 Hz, 2H), 6.99–6.95 (m, 3H), 6.89 (d, *J* = 2.4 Hz, 1H), and 5.10 (s, 1H). ^13^C NMR (100 MHz, Acetone-d6) δ 178.1, 169.0, 163.2, 156.9, 156.3, 145.2, 127.8, 122.8, 117.4, 116.8, 115.4, 103.3, and 89.4.

##### 2-(4-Fluorophenoxy)-7-hydroxy-chromen-4-one (**12b**)

An amount of 100.0 mg (0.35 mmol) of **6c** was dissolved in anhydrous DCM (1.5 mL) and then treated with BBr_3_ (1 M in DCM, 1.1 mL) dropwise at 0 °C in an inert atmosphere. After 1 h had passed, the resulting solutions were continuously stirred at ambient temperature overnight, and the reaction mixture was diluted with water (10 mL) and partitioned with ethyl acetate (10 mL × 3). The organic phases were combined and concentrated in vacuo. The crude product was purified by column chromatography (silica gel, dichloromethane: ethyl acetate = 5:1, *v*/*v*) to produce **12b** as a white solid (90.1 mg, yield: 94.6%).^1^H NMR (400 MHz, Acetone-d6) δ 9.59 (s, 1H), 7.91 (d, *J* = 8.8 Hz, 1H), 7.46–7.41 (m, 2H), 7.35–7.30 (m, 2H), 6.98 (d, *J* = 8.8, 2.0 Hz, 1H), 6.89 (d, *J* = 2.0 Hz, 1H), and 5.17 (s, 1H). ^13^C NMR (100 MHz, Acetone-d6) δ 178.0 (C-4), 168.0 (C-2), 163.3 (C-7), 161.6 (d, *J* = 242.0 Hz, C-4′), 156.4 (C-9), 148.9 (C-1′), 127.9 (C-5), 123.7 (d, *J* = 9.0 Hz, C-2′, 6′), 117.9 (d, *J* = 24.0 Hz, C-3′,5′), 116.8 (C-10), 115.5 (C-5), 103.3 (C-8), and 90.3 (C-3). HRMS [ESI]^+^ calculated for [C_15_H_9_FO_4_ + H]^+^ 273.0558; found [M + H]^+^ 273.0559.

### 3.2. Cell Line and Cell Culture

Huh7 cells (RRID: CVCL_0336) were cultured with Dulbecco’s modified minimal essential medium (DMEM), supplemented with 10% FBS, P/S (penicillin (100 U/mL) and streptomycin (100 μg/mL)), nonessential amino acids (0.1 mM), and L-glutamine (2 mM) (Thermo Fisher Scientific, Waltham, MA, USA) at 37 °C with 5% CO_2_.

### 3.3. Lipid Droplets (LD) Assay

An LD assay was performed as described in previous research [36]. The accumulation of LDs was detected with the BODIPY^®^ 493/503 dye (Thermo Fisher Scientific). LD accumulation resulted from treating cells with bovine serum albumin (BSA)-conjugated oleic acid (OA). Cells were seeded in μClear^®^ 96-well plates (Greiner Bio-ONE, Frickenhausen, Germany) and loaded with 125 μM of OA with testing compounds at a concentration of 40 μM for 16 h. Cells were then fixed with paraformaldehyde and stained with 2 μg/mL of Hoechst 33342 and 1 μg/mL of BODIPY^®^ 493/503. Nine fields for each well were picked, and images of nuclei and LD were acquired and analyzed automatically with an HCS instrument (ImageXpress Micro System, Molecular Devices, Sunnyvale, CA, USA). A granularity analyzing module was used to identify nuclei and LDs. The diameter settings for defining nuclei and LDs were 8–25 and 0.5–2 μm, respectively. The average LD counts/cell of BSA-conjugated OA + drug vehicle (DMSO)-treated wells (hereinafter referred to as OA) were used as the standard for 100% of fatty loading.

### 3.4. RNA Isolation, Reverse Transcription (RT), and Real-Time PCR (qPCR)

Total RNA was isolated from cells using TRIzol (Life Technologies, Carlsbad, CA, USA) according to the manufacturer’s protocol. Complementary DNA (cDNA) was produced from cellular RNA (1 μg) using a high-capacity cDNA reverse transcription kit (Thermo Fisher Scientific). Real-time PCR reactions were performed using a TOOLS Easy SYBR qPCR Mix (TOOLS, Taipei, Taiwan). Reactions were assayed using an Applied Biosystems StepOnePlus Real-Time PCR system. The mRNA level was normalized with the TATA box binding protein (TBP) mRNA level. The primer pairs used are shown in Table 2.

### 3.5. Statistical Analyses and Hit Selection

All data were analyzed with GraphPad Prism 5.01 software (La Jolla, CA, USA). One-way analysis of variance (ANOVA) followed by Dunnett’s comparison test was used to compare differences between multiple groups. A *p*-value < 0.05 was considered statistically significant. In the process of hit selection, compounds that were capable of reducing LD formation by >40% (LD content <60%, which was approximately equivalent to mean-2.5SDs (mean and SD of all tested compounds)) without severe cytotoxicity (cell viability >85%, which was approximately equivalent to mean-0.5SDs of all tested compounds) were considered as screening hits.

## 4. Conclusions

In this study, a series of 2-phenoxychromone derivatives were designed and synthesized, and their ability to reduce the amount of lipid droplets in Huh7 cells was evaluated. Among these derivatives, those with trimethoxy groups at the phenyl moiety exhibited significant activity. Moreover, compound **7e** was identified as a potential agent with an IC_50_ value of 32.2 ± 2.1 μM against lipid accumulation and no significant cytotoxicity. Upregulation of PGC1α gene expression in **7e**-treated groups suggested that **7e** facilitated the catabolism of lipid in Huh7 cells. In conclusion, this newly developed 2-phenxoychromone derivative can reduce lipid accumulation in hepatocytes and shows potential as a therapeutic agent in managing NAFLD or NASH.

## Figures and Tables

**Table 1:** Relative lipid droplet count and relative nuclear count in Huh7 cells treated with 2-phenxoychromones.

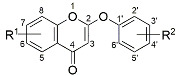				
Compound	R^1^	R^2^	Relative Lipid Droplet Count ^a^	Relative Nuclear Count ^b^
**4a**	5-OCH_3_	H	N/A ^c^	N/A ^c^
**4b**	5-OCH_3_	4′-OH	139.9 ± 8.5	108.2 ± 12.9
**4c**	5-OCH_3_	4′-F	N/A ^c^	N/A ^c^
**4d**	5-OCH_3_	4′-OCH_3_	118.4 ± 11.0	100.7 ± 20.6
**4e**	5-OCH_3_	3′,4′,5′-OCH_3_	93.7 ± 7.5	100.8 ± 7.7
**5a**	6-OCH_3_	H	109.6 ± 6.6	93.4 ± 9.4
**5b**	6-OCH_3_	4′-OH	118.5 ± 2.9	99.7 ± 6.3
**5c**	6-OCH_3_	4′-F	93.1 ± 5.6	88.5 ± 8.7
**5d**	6-OCH_3_	4′-OCH_3_	93.1 ± 5.1	84.8 ± 4.7
**5e**	6-OCH_3_	3′,4′,5′-OCH_3_	59.6 ± 5.3	58.7 ± 4.7
**6a**	7-OCH_3_	H	141.3 ± 7.9	92.1 ± 8.2
**6b**	7-OCH_3_	4′-OH	129.4 ± 4.8	105.5 ± 10.3
**6c**	7-OCH_3_	4′-F	131.2 ± 7.4	110 ± 16.7
**6d**	7-OCH_3_	4′-OCH_3_	153 ± 10.8	114.8 ± 15
**6e**	7-OCH_3_	3′,4′,5′-OCH_3_	111.1 ± 16.5	108 ± 15.7
**7a**	5,7-OCH_3_	H	151.5 ± 4.9	105.8 ± 11.8
**7b**	5,7-OCH_3_	4′-OH	90.2 ± 4.1	100 ± 16.1
**7c**	5,7-OCH_3_	4′-F	123.7 ± 10.2	104.7 ± 11
**7d**	5,7-OCH_3_	4′-OCH_3_	105.6 ± 5.8	109 ± 16.9
**7e**	5,7-OCH_3_	3′,4′,5′-OCH_3_	59.6 ± 4.1	103.1 ± 6.5
**8a**	5,6,7-OCH_3_	H	106.7 ± 6.8	102.6 ± 10.8
**8b**	5,6,7-OCH_3_	4′-OH	99.1 ± 4.8	105.4 ± 6.6
**8c**	5,6,7-OCH_3_	4′-F	107.5 ± 7.2	100.1 ± 10.1
**8d**	5,6,7-OCH_3_	4′-OCH_3_	108.9 ± 7.5	100.7 ± 12.3
**8e**	5,6,7-OCH_3_	3′,4′,5′-OCH_3_	77.8 ± 8.6	118.8 ± 13.8
**9a**	7-F	H	116.7 ± 5	98.2 ± 14.8
**9b**	7-F	4′-OH	90.6 ± 7.0	95.6 ± 9.0
**9c**	7-F	4′-F	126.9 ± 4.3	111.2 ± 14.1
**9d**	7-F	4′-OCH_3_	112 ± 8.3	92.4 ± 17.4
**9e**	7-F	3′,4′,5′-OCH_3_	92.7 ± 3.6	108.8 ± 6.7
**10a**	5-OH	4′-OH	125.9 ± 7.9	63.2 ± 22.3
**10b**	5-OH	4′-F	122.8 ± 7.4	69.1 ± 10.0
**11a**	6-OH	4′-OH	108 ± 6.2	99.9 ± 7.7
**11b**	6-OH	4′-F	119.1 ± 5.0	103.7 ± 13.4
**12a**	7-OH	4′-OH	89.3 ± 5.1	92.0 ± 8.8
**12b**	7-OH	4′-F	135.1 ± 4.7	84.3 ± 8.4
**Capillarisin**	5,7-OH, 6-OCH_3_	4′-OH	104.4 ± 8.0	74.6 ± 8.7

^a^ Lipid droplet count: The average LD counts/cell of OA (BSA-conjugated OA + DMSO) were used as standard for 100 % of fatty loading in Huh7 cell line. The pure compound concentration is 40 µM. ^b^ Relative nuclear count: The average nuclear counts/cell of OA (BSA-conjugated OA + DMSO) were used as standard for 100 % of fatty loading in Huh7 cells. ^c^ Easily decomposed under normal storage conditions.

**Table 2:** Primers used in this study.

Gene	Species	Forward (5′ > 3′)	Reverse (5′ > 3′)
*CD36*	*Homo sapiens*	TCCTGCAGAATACCATTTGATCC	GGTTTCTACAAGCTCTGGTTCTTA
*CPT1*	*Homo sapiens*	TCCAGTTGGCTTATCGTGGTG	CTAACGAGGGGTCGATCTTGG
*DGAT1*	*Homo sapiens*	CAACAAGGACGGAGACGCCGG	GATGCCACGGTAGTTGCTGAAGCC
*APOB*	*Homo sapiens*	ACCTCCAGAACATGGGATTGC	GGGCTGGTGTCCTAACAGTC
*MTTP*	*Homo sapiens*	TGAGGCAGTGGCCATAGAAAAT	CTTTGTCTTGATGAGCCTGGTA
*PGC1a*	*Homo sapiens*	GTCACCACCCAAATCCTTAT	ATCTACTGCCTGGAGACCTT
*TBP*	*Homo sapiens*	CAGAAGTTGGGTTTTCCAGCTAA	ACATCACAGCTCCCCACCAT

## Data Availability

The authors confirm that the data supporting the findings of this study are available within the article and its supplementary materials.
